# Development of a new class of stable and adaptable free-standing fibre mats with high room-temperature hydroxide-ion conductivity

**DOI:** 10.1038/s41598-024-64646-9

**Published:** 2024-06-24

**Authors:** Servann Hérou, Pauline Kasongo-Ntumba, Arun Prakash Periasamy, James King, Molly McVea, Szymon Doszczeczko, Andy Bushby, Ana Belen Jorge Sobrido, Maria-Magdalena Titirici, Petra Ágota Szilágyi

**Affiliations:** 1 Department of Chemical Engineering, Imperial College Road, London, SW7 2AZ UK; 2 School of Engineering and Materials Science and Materials Research Institute, Queen Mary University of London, Mile End Road, London, E1 4NS UK; 3 Department of Chemistry, SRM Institute of Science and Technology, Kattankulathur, Tamil Nadu 603 203 India; 4 Department of Chemistry, Centre for Materials Science and Nanotechnology (SMN), University of Oslo, Blindern, P.O. Box 1033, 0315 Oslo, Norway

**Keywords:** Coordination chemistry, Electrochemistry, Energy, Materials chemistry

## Abstract

For alkaline anion-exchange membrane electrolysers and fuel cells to become a technological reality, hydroxide-ion (OH^−^) conducting membranes that are flexible, robust, affording high OH^−^ conductivity, and synthesised in a low-cost and scalable way must be developed. In this paper, we engineer a stable, self-supporting, and flexible fibre mat using a low-cost ZIF-8 metal–organic framework composited with ionic liquid tetrabutylammonium hydroxide and widely used polyacrylonitrile as polymeric backbone. We obtain mats with a high intrinsic OH^−^ conductivity for a metal–organic framework-based material already at room temperature, without added ion-conductor polymers. This approach will contribute to the development of low-cost and tuneable ion-conducting membranes.

## Introduction

Hydrogen is a promising energy vector, however for its full-scale deployment, several technological and infrastructural barriers must be overcome. One of these relate to electrochemical devices in which hydrogen is formed (electrolyser) or converted to water (fuel cell). Alkaline-anion-exchange membrane water electrolysers (AAEMWE) and fuel cells (AAEMFC)^1,2^ are promising technologies as they may operate with earth-abundant electrocatalysts, however, their large-scale commercialisation has to date been hindered by the development of suitable anion exchange membranes. These AAEMs must exhibit the following properties: (i) good mechanical and chemical stability to avoid long-term degradation in commercial electrolytes; (ii) high OH^−^ conductivity to ensure high power densities and efficiencies when assembled in full devices, and (iii) scope to be synthesised in an economically viable and scalable route. Unlike Nafion^®^ that is widely used in commercially available proton exchange membrane fuel cells (PEMFCs)^3–5^, there is yet no benchmark for AAEMs (although the performances of commercially available AAEMs such as Fumasep^®^ FAA3-50 and Sustanion are often compared in the literature, more detailed investigation of their characteristics is still needed to be considered benchmark). Highly conductive membranes (> 50 mS cm^−1^ at 20 °C) have been recently synthesised on a laboratory level (< gram/batch) but these materials are challenging, energy and/or time-consuming to synthesise, and very expensive to produce on a larger scale^6,7^.

Metal–organic frameworks (MOFs) offer several advantages in terms of ion conductivity^8^. Firstly, most of them are electrical insulators, which is an essential property for ion-exchange applications. Secondly, they are highly porous, which facilitates efficient mass, including ion transport. Finally, they are highly tuneable both structurally and chemically so they can be optimised to offer bespoke solutions to particular applications and challenges. The stability and cost of MOF-based membranes are usually considered as the bottleneck to their commercialisation, however, there has been a tremendous advance on both issues, with the development of MOFs which are not only stable in aqueous media but also have applications in water capture in harsh environments^9,10^, while low-cost base-metal precursors and cheap organic building blocks (e.g. terephthalates and imidazoles), and increasing demand continue to lower their manufacturing costs^11^. These materials are often brittle and must be used on substrates (i.e. polymeric gels and fibres) to improve their flexibility^12–21^. Such composite membranes have shown remarkably good performances in PEMFCs with high proton conductivities reaching up to 76 mS cm^−1^ at 25 °C and 100% relative humidity, albeit the polymer used often affords high intrinsic ion conductivity on its own^22^.

To reach such high OH^−^ conductivity values in the same operating conditions, MOF-based films typically are grafted or otherwise combined with an intrinsically OH^−^ containing ionic compound, which we refer to as doping. This strategy allows for (i) confinement of the ions in the MOF pores, which prevents cation leaching when in contact with the electrolyte and turns the diffusion layer into a compact layer, accelerating the charge-transfer kinetics in the MOF nanopores^23^; (ii) improvement of the hydrophilicity of the pores, which increases water uptake capacity and facilitates the OH^−^ diffusion by the fast Grotthus mechanism with proton back-donation^6,24–26^. For instance, FJU-66 doped with [EVIm]OH ionic liquid (IL) reaches a very high 91 mS cm^−1^ ion conductivity at 85 °C but its performance drops to 60 mS cm^−1^ at 30 °C^27^. In contrast, at room temperature, ZIF-8 doped with choline hydroxide only shows an ion conductivity of 4·10^−4^ mS cm^−1^^12^. Poly-ionic liquids and interpenetrated conducting polymers can also be encapsulated within the MOF structure which provides high OH^−^ ion conductivities reaching 30–70 mS cm^−1^ at 30 °C^19,20^. However, these recent developments in MOF-based composites often display the highest ion conductivity at high temperatures (> 80 °C), typically combined with ion-conducting polymers, and they do not address issues related to the mechanical robustness and flexibility of the membrane, two factors that are also crucial for enabling device integration and hence application^16,28^.

This work features the processing of the zeolitic imidazolate framework ZIF-8, into a self-supporting flexible membrane using electrospinning for its simultaneous integration and nano-structuring into a robust and flexible nanofibrous mat. ZIF-8 was selected as host for hydroxide ions for its stability in alkaline environment, its commercial availability, and its building blocks and precursors (Zn and methylimidazole) being abundant and low-cost^29–32^. We carried out the electrospinning in a well-established route employing the inexpensive polyacrylonitrile (PAN) substrate backbone, which acts as an insulating, flexible and resilient scaffold to support the ZIF-8 crystallites. To improve the intrinsic OH^−^ conductivity of the fibre mat, the MOF particles were subsequently doped with tetrabutylammonium hydroxide (TBAH) IL, through anchoring the cations into the MOF cages, thereby loosening their interaction with the now nanoconfined hydroxide ions. This doping effect has been previously shown to improve the ion conductivity in the polycrystalline MOF^26^ but has not yet been implemented into a self-supporting film. It is hereby shown that engineering the MOF shape and microstructure does not only allow for better mechanical properties, but also for a partial organisation of the MOF crystallite packing, which drastically boosts the conductivity. As a proof of concept, we measured OH^−^ conductivity for the fibre mats at room temperature under humidified inert atmosphere to show their potential in low-temperature operated alkaline water electrolysers and fuel cells (operating at temperatures from 20 °C), with additional perspective in water desalination and capacitive deionisation.

## Results and discussion

With the ultimate goal of device integration (albeit outside of the scope of this work), it should be recognised that ion-conducting films must combine mechanical and chemical robustness with accessible porosity to enable fast ion diffusion while demonstrating the potential for low-cost production. As the processing itself should also be cost-effective and scalable, electrospinning stands out as a competitive production route to affording flexible fibre mats with tuneable porosity and surface chemistry^33^. In order to balance the brittleness of MOFs with the low electrical conductivity of the PAN polymer backbone, we electrospan both compounds simultaneously using the following PAN:ZIF-8 mass ratios: 10:6, 10:8, and 10:10. This way, the high elastic modulus of PAN nanofibres creates a robust scaffold for the ZIF-8, which in turn hosts and mobilises the hydroxide ions. In addition to being commercially viable, PAN is also conveniently insoluble in polar solvents, which facilitates the fibre mat synthesis. ZIF-8 was obtained following a solvothermal reaction between Zn(NO_3_)_2_·6H_2_O and 2-methylimidazole in methanol, adapted from a procedure outlined in the literature (see Materials and Methods)^26^.

As the bare PAN:ZIF-8 composite fibre mats exhibit intrinsic OH^−^ conductivities limited to 10^−3^ mS cm^−1^, tetrabutylammonium hydroxide was immobilised within the micropores of the ZIF-8 using an approach first developed by Sadakiyo et al*.*^26^, increasing both the hydrophilicity and intrinsic OH^−^ content of the framework in a bid to improve ion conductivity. This was achieved by immersing the fibre mats in a methanolic solution of TBAH at 60 °C for 24 h. Their stability was then tested by soaking them for 10 days in a 2 M methanolic sodium hydroxide solution prepared using anhydrous MeOH and degassed NaOH. Methanol was used as solvent in a bid to minimise the in-situ formation of carbonate ions (CO_3_^2−^) which have been shown to drastically reduce OH^−^ diffusion^26^. These last samples will be henceforth referred to as ‘aged’ samples. The ion conductivities of the membranes were tested on the as-spun composites, their TBAH-doped analogues as well as on the aged samples.

**Figure 1:**
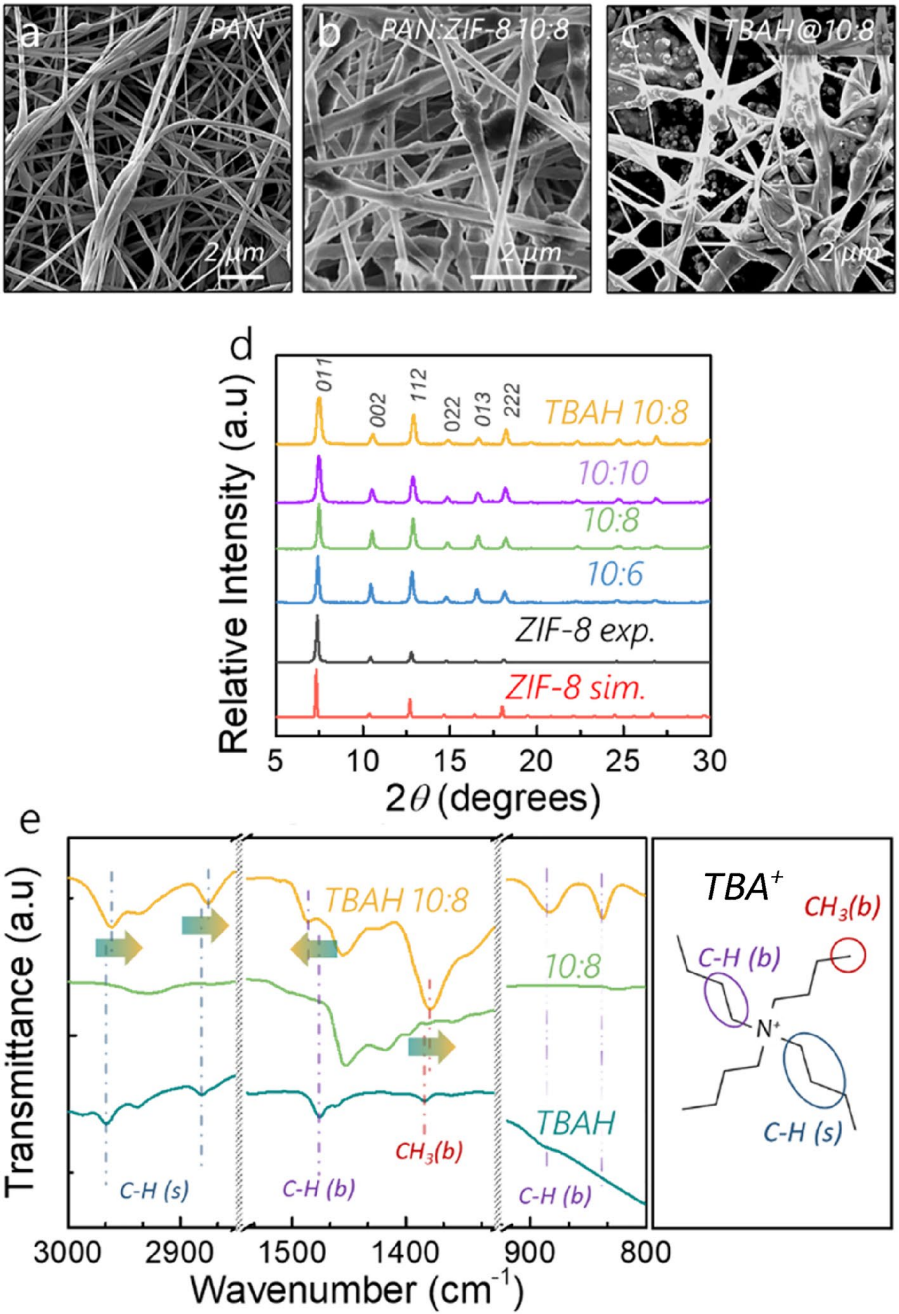
Scanning electron micrographs of (**a**) pure PAN, (**b**) PAN:ZIF-8 10:8, (**c**) TBAH@PAN:ZIF-8 10:8, (**d**) PXRD patterns showing the relative intensity of simulated ZIF-8, synthesised ZIF-8, PAN:ZIF-8 with ratios of 10:6, 10:8 and 10:10 and TBAH@PAN:ZIF-8 10:8. The crystallographic peaks were labelled as in the literature and all normalised to (011)^32^; (**e**) Fourier-Transformed Infrared normalised spectra of pure TBAH, PAN:ZIF-8 10:8, and TBAH@PAN:ZIF-8 10:8. The annotated peaks in the TBAH@PAN:ZIF-8 spectrum correspond to those also found in the TBAH spectrum that indicate that TBA^+^ is immobilised inside the ZIF8 pores. Full FTIR spectra can be found in Figs. S2 and S3.

The combination of ZIF-8 with PAN does not significantly affect the morphology and microscopic surface of the nanofibre mats (Fig. 1a,b), it nonetheless results in a slight, 20%, diameter increase (Fig. S1). The additional Zn signal in X-ray photoelectron spectroscopy (XPS, Fig. S2) evidences the successful compositing of the MOF with PAN. In addition to the homogeneous dispersion of ZIF-8 throughout the fibre, in line with what was previously observed in the literature^14^, the electrospinning also maintains the crystal structure of the ZIF-8, as demonstrated by the resultant powder X-ray diffractograms (PXRD, Figs. 1d and S4a–c). When compared with the diffractogram collected on powdered ZIF-8 (identical to the simulated trace), the composite fibres however show a relative intensity increase of the of the (002) Bragg peak with respect to other ones, such as the (011) peak. On closer inspection of the crystal structure, it can be observed that, unlike the (002) plane, the (011) plane does not travel through the MOF cages (Fig. S3). This implies that a degree of particle orientation across the pores becomes relatively more abundant upon electrospinning, which is consistent with an alignment of channels along the nanofibres^30,32,34^. Such particle alignments have been previously observed using electrospinning and have been rationalised as induced by the polarisation of the particles and lattice mobility during spinning^35^. Most importantly, Sadakiyo et al*.*^26^ have refined the TBAH-doped ZIF-8 structure to show that the TBAH guest is located in the ZIF-8 cage, which is traversed directly by the (002) plane. As hydroxide ions are interacting with the framework electrostatically, their strongest-binding adsorption site, and consequently their highest intrinsic concentration, must be in the vicinity of positive charges, i.e. also in the large ZIF-8 pores. This means that the intrinsic hydroxide-ion content, and thus its conduction pathway, is associated with this particular crystallographic plane, and so long as the OH^−^ conductivity takes place intrinsically (i.e. within the MOF structure), it should be affected by the crystallites’ orientation involving the (002) plane. It therefore stands to reason that the increased relative occurrence that we observe in this direction should also affect the hydroxide-ion conductivity of the MOF-composite fibres when compared with the randomly oriented powder sample.

After doping, thorough washing and drying of the fibre mats, the presence of TBA^+^ ions were evidenced through the emergence of an additional peak in the N 1*s* XPS spectrum (Fig. S2). As previously suggested, TBA^+^ likely enhances OH^−^ conductivity via increasing the hydrophilicity of the ZIF-8 framework. This greater hydrophilicity can be rationalised in terms of hydrophobic-hydrophobic interactions between the butyl groups of the TBA^+^ and the methyl groups present in ZIF-8 linkers, conferring an intrinsic electric charge to the MOF framework, which in turn increases its affinity towards water adsorption^26^. The higher hydrophilicity of the doped samples is also confirmed by the appearance of a small additional O 1*s* peak in the XPS spectrum (Fig. S2), which can be likely attributed to hydroxide ions and/or water molecules in the material.

The diffractogram reveals that the ZIF-8 topology is preserved upon doping by TBAH-loading into the pores (Fig. 1d), However, a closer examination also shows an intensity-ratio change of some Bragg peaks as the intensity of the (002) and (013) peaks decreases significantly. As seen before, using the model developed by Sadakiyo et al*.*^26^, in which the tetrabutylammonium ion is located inside the pore, it is apparent that both (002) and (013) planes cross the TBA^+^ ion, i.e. the electron density through these planes increases on the addition of the guest TBAH. This increase of electron density lowers the scattering contrast between pores and the walls resulting in the specific intensity decrease of Bragg peaks corresponding to the crystallographic planes that traverse the pores, e.g. (002), (013), compared with those, which do not, e.g. (112)^36^. This demonstrates that the TBAH is grafted inside the pores, which is in good agreement with the Fourier-Transform Infrared (FTIR) spectroscopy results also showing strong framework-guest interactions for the TBAH doped PAN:ZIF-8 10:8 (Figs. 1e and S4d–f). The spectrum of the TBAH-doped PAN:ZIF-8 fibre mat indeed exhibits the characteristic C–H stretching (*s*) modes of the butyl groups in the bulk TBAH at 2962 and 2875 cm^−1^, which are not present in the undoped mat. The two peaks are however slightly redshifted in the doped sample compared with the bulk substance, as it is observed in the literature for the case of confined ions^37^. Similarly, the C–H bending mode (*b*) at 1475 cm^−1^ is also shifted on guest grafting. The broad features around 830 and 880 cm^−1^ in the bulk FTIR spectrum of TBAH, may also be assigned to C–H bending modes. Interestingly, these two bands appear much more sharply at 837 and 883 cm^−1^ in the doped sample, we tentatively attribute this spectral sharpening to the more defined molecular interactions on account of a less extended hydrogen-bonding network present in the doped sample, compared with the pure IL. On account of its low vapour pressure and the strong capillary forces maintained between the nanofibres, it cannot be excluded that TBAH still fills some narrow interfibrous voids (Figs. 1c and S2). In addition, such unanchored TBAH should contribute to ion conductivity. However, we would like to emphasise that we do not observe any broadening of the signals arising from the TBAH, which could be interpreted as a multitude of similar species or those interacting with their surroundings to different strength. In addition, the C–H signals, both *s* and *b*, shift on combining TBAH with ZIF-8. This is important, as previously it has been suggested that the tetrabutylammonium ion interacts with the methyl group of the MOF linker^26^, which theory is underpinned by our above observations. If there were a significant amount of TBAH outside of the MOF pores, it would be in its bulk form and would not interact strongly with the framework (no confinement effect), which also means that such species would result in FTIR spectral features identical to the bulk, in addition to those shifted on account of the hydrophobic host–guest interactions. As we do not see such signals or such convolution, we conclude that the vast majority of TBAH is indeed confined in the ZIF-8 pores, and any ion conductivity through it should therefore be linked to the OH^−^ within the MOF channels, as not only will be the vast majority of ionic species bound within the channels.

The fibre mats’ robustness was evaluated by nanoindentation measurements (Fig. S6) to gauge their future potential for integration in a device. In the used electrospinning configuration, the mats are produced by random deposition of nanofibres on the collector. Consequently, nanoindentation probes the compressibility of the mats (rather than the modulus of the individual fibres), which exhibits an elastic modulus obtained for the pure PAN nanofibre mats of 50 kPa. The addition of ZIF-8 into the PAN nanofibres (10:6, 10:8), does not significantly affect the Young moduli (*E*) of the specimens, which remain around 30 kPa. At higher ZIF-8 loadings (10:10), a greater continuity in the ZIF-8 structure may explain an increase of *E* to 0.1 MPa, as the pure ZIF-8 shows typical values around 3 GPa^38^. Interestingly, doping the 10:10 PAN:ZIF-8 sample with TBAH further increased *E* to 0.5 MPa, as the presence of the cation inside the MOF channels likely effects stress on the crystal, thus reducing its plasticity. Even after the addition of ZIF-8 in the nanofibres, the mats maintain the high elasticity obtained for the bare PAN mats, which highlights the promise of this processing approach.

As expected, the presence of TBAH in the ZIF-8-based composite membranes influences their OH^−^ ion conductivities. The ion conductivities in terms of OH^−^ ions conduction were determined via charge-transfer resistance using converging methods: (i) the linear regression of the Nyquist plots, which simulate the combination of resistive and capacitive processes (see ESI Materials Characterisation, Figs. S7 and S8); (ii) the calculation of the impedance modulus, |*Z*|, at the maximum phase angle *θ*_max_, where the system transits from an electrically governed conductivity to an ionically governed one at lower frequencies. Therefore, the effects of ZIF-8 integration, TBAH doping and ageing in NaOH on the ion conductivity are demonstrated through the investigation of the specimen PAN:ZIF-8 10:8 as it shows the highest performances. The Nyquist plots are shown in SI (Fig. S7), for the sake of simplicity, all composite mats are not presented herein.

**Figure 2:**
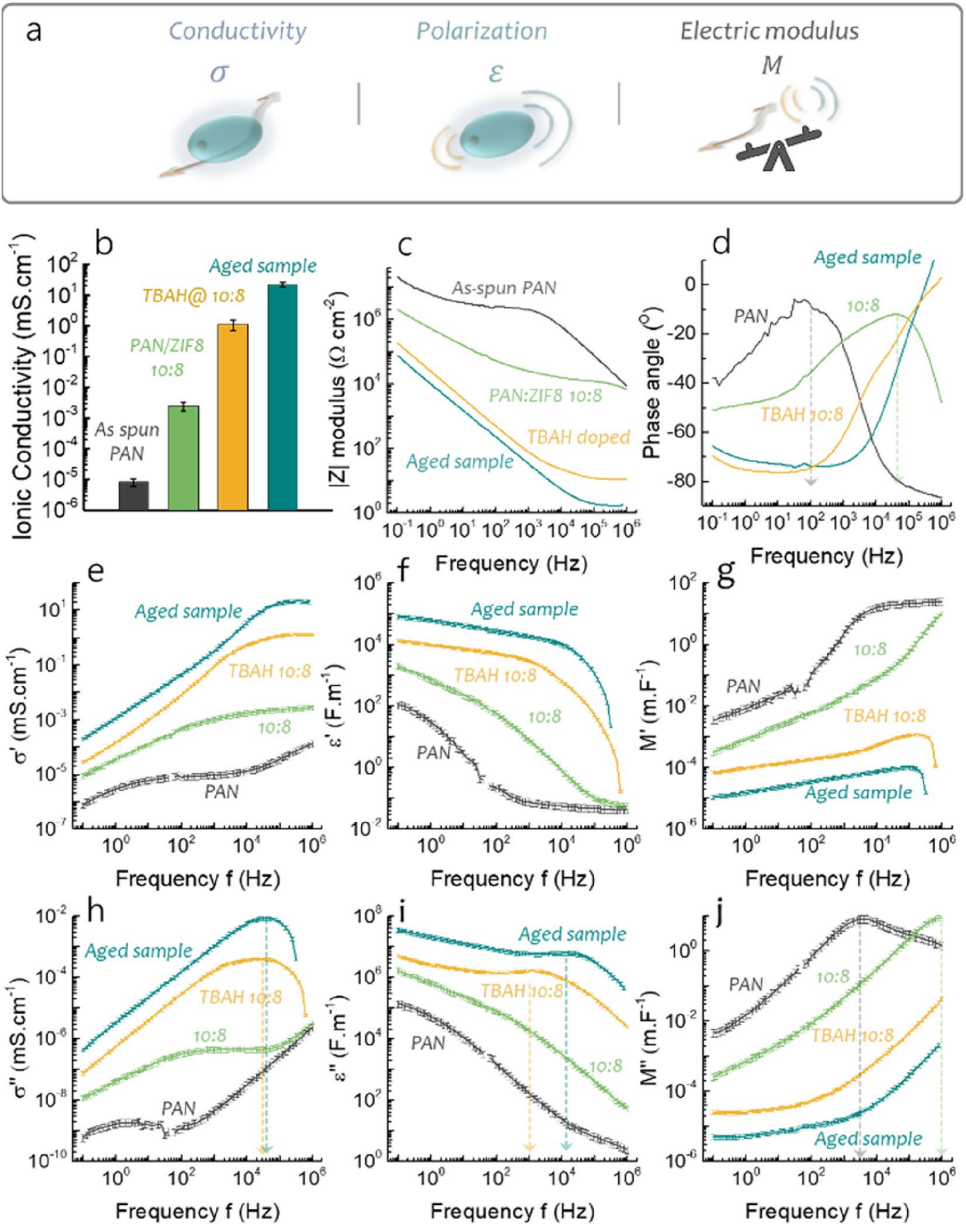
(**a**) Schematic of the physical meaning of conductivity, permittivity and electric modulus; (**b**) Ionic conductivity obtained from charge transfer measurements at 22 ± 3 °C and relative humidity of 95% $\pm 3\%$; (**c**) Areal *Z* modulus |*Z*| (Ω cm^−2^); (**d**) Phase angle between the current observed and the voltage applied; Real component of the electrical (**e**) Real conductivity $\sigma^{\prime }$; (**f**) Real permittivity $\varepsilon^{\prime }$; (**g**) Real electric modulus $M^{\prime }$; (**h**) Imaginary conductivity $\sigma^{\prime \prime }$; (**i**) Imaginary permittivity $\varepsilon^{\prime \prime }$; (**j**) Imaginary electric modulus $M^{\prime \prime }$ with related error bars for the as-spun PAN membranes, the undoped and doped PAN:ZIF-8 10:8 as well as the aged membranes. Imaginary components are shown on Fig. S12.

The poor ion conductivity of the as-spun PAN (8.3·10^−6^ mS cm^−1^) is increased by over two orders of magnitude upon addition of ZIF-8 to reach 2·10^−3^ mS cm^−1^ (Fig. 2b). This clearly indicates a more efficient ion migration through the ion-conducting channels, afforded by the porous ZIF-8 framework rather than through the PAN. In comparison, the pristine ZIF-8 powders show OH^−^ conductivities in the range of 10^−9^ mS cm^−1^^26^, which also indicates that the preferred orientation of the ZIF-8 crystallites during electrospinning plays a role in the ion diffusion. At this stage, the hydroxide ions (radius: 1.10 Å) are derived from the autoprotolysis of water at high relative humidity and their transport is facilitated by the open pores (11.6 Å) and small apertures (3.4 Å) present in the ZIF-8 crystals coupled with dehydration effects due to nano-confinement^23,39,40^. After doping the ZIF-8 framework with TBAH, the ion conductivity further increases from 2·10^−3^ mS cm^−1^ to 1 mS cm^−1^ mainly due to increased hydrophilicity of the pores on account of the additional electrically charged species, which in turn facilitates the mobility of OH^−^ (Fig. 2b).

To explore the mechanism of ion conduction through the mats, we probed both long-range ($f\to 0$) as well as short-range ($f\to \infty$) ion dynamics. The frequency dependence of the complex impedance modulus (*Z*), conductivity (*σ*), permittivity (*ε*) and electric modulus (*M*) were analysed by Electrochemical Impedance Spectroscopy (EIS) (see Materials and Methods, Eqs. 5–11)^41^. The real electric modulus $M^{\prime }$ relates to the balance between conductive and dielectric effects while $M^{\prime \prime }$ describes the transition from a long-distance mobility observed at low frequencies to a short-distance mobility at high frequency where the charge carriers are confined by the local ionic potential wells that prevent successful hopping mechanisms (Fig. 2a)^42^.

The frequency range studied (10^−1^ to 10^6^ Hz) relates to rather slow time-scale processes such as the segmental motion of the polyacrylonitrile polymer chains, the vibrations of the ZIF-8 as well as the migration of ions in the composite material (polymer or MOF)^43^. The fibre mats exhibit the characteristics of poor conductors at low frequencies due to the absence of conjugated domains in the polyacrylonitrile and ZIF-8. At lower frequencies, the slow oscillation of the electric field creates a strong polarisation at the electrodes surface due to the long-distance diffusion of the mobile charge carriers. This translates into large impedance moduli values beyond 10^5^ Ω cm^−2^ below 10 Hz (Fig. 2c), as is the case for the electrospun PAN mat which exhibits the lowest conductivity $\sigma^{\prime }$ and dielectric constant $\varepsilon^{\prime }$ (Fig. 2e,f). The addition of ZIF-8 into the PAN polymeric network improves the conductivity ten-fold, in agreement with the expected higher electrical conductivity of the ZIF-8 compared with PAN (Fig. 2e)^44,45^. This difference in conductivity can be explained by the combination of the orbitals of the linkers (methylimidazole) and the metal nodes (Zn^2+^) in the ZIF-8 affording a better conduction pathway through the ZIF-8 framework, compared to the PAN polymeric backbone, which displays strong covalent C≡N bonds with low energy HOMO^45,46^. This also explains the increase in the dielectric constant, $\varepsilon^{\prime }$, because the composite nanofibres become more polarisable. As the frequency increases, the dipoles progressively relax as the polar groups in the polymer and the ZIF-8 are unable to reach a new equilibrium within a single oscillation of the electric field. The highly mobile charge carriers (i.e. ions) can diffuse through the sample over much shorter distances and the potential bulk diffusion effect disappears. In the absence of OH^−^ doping, the conduction of hydroxide ions through the specimen is facilitated by the high humidity providing ionic species in extremely low concentrations (with the sole source being water autoprotolysis)^47^. As the excitation frequency increases, the polymer chains relax and the ion conductivity becomes detectable^43^. The high electric modulus $M^{\prime }$ in both the pure PAN and the composite PAN:ZIF-8 suggest that ionic polarisation effects dominate over ion conductivity (Fig. 2g). The absence of charged coordination sites in the as-spun PAN and PAN:ZIF-8 10:8 composite membranes, as well as the short life-time of H_3_O^+^ and OH^−^ ions from the water condensed into the ZIF-8 channels in the high RH conditions^48^, limit the conductivity of the PAN:ZIF-8 10:8 composite membranes to 10^−3^ mS cm^−1^ (Fig. 2e)^47^. Upon addition of the ZIF-8, the $M^{\prime \prime }$ peak shift indicates a longer ionic diffusion range achieved by successful hopping mechanism and thus a more effective conduction process is achieved (Fig. S2j)^42^. A similar peak shift observed for the dielectric loss, $\tan \left( \delta \right)$, suggests that the addition of ZIF-8 increases the relaxation frequency of the material, due to its much higher polarisability (Fig. S8b). The compositing of ZIF-8 within PAN nanofibres therefore has a positive impact on the ion transport, which is confirmed by the shift in the maximum phase angle towards high frequency values (Fig. 2d). Due to long-range ion transport below 10^6^ Hz, the ion kinetics is partially diffusion-limited and the phase angle *Φ* remains around − 45°. This could be related to ionic aggregation and reduced polymer segmental motion resulted from enhanced polymer–polymer interactions.

By doping the fibre mats with TBAH, the ion conductivity increased by a factor of 10^4^ to reach 1 mS cm^−1^ (Fig. 2e) at 20 °C. The presence of highly mobile ionic species (i.e. OH^−^) is laid bare as the |*Z*| modulus drops and the phase angle *Φ* (Fig. 2d) shifts from a rather resistive system in the undoped fibres (*Φ* > − 20°) to a rather capacitive system (*Φ* < − 60°) below 10^4^ Hz. This suggests that the presence of TBA^+^, previously shown to be immobilised into the cages of the ZIF-8 (Fig. 1c,d), improves the hydrophilicity of the material and confines the diffusive layer into a compact layer, allowing much faster OH^−^ hopping (i.e. Grotthuss mechanism^6,24–26^) between the water molecules confined within the ZIF-8 channels^26,49^. This increased charge-carrier concentration in the ZIF-8 induces a capacitive behaviour, also characterised by an increased dielectric constant, $\varepsilon^{\prime}$, upon doping. As the frequency increases, $\varepsilon^{\prime}$ decreases drastically and $M^{\prime }$ remains relatively constant indicating that the charge-transfer contribution dominates over polarisation effects (Fig. 2f,g). The ion conductivity $\sigma^{\prime}$ increases indeed from 0.1 mS cm^−1^ at 10^3^ Hz to 1 mS cm^−1^ at 10^5^ Hz. This shift in behaviour is clearly visible as the peak in the $M^{\prime \prime }$ spectra is shifted to even larger frequencies, confirming that the ion conductive response to $\vec{E}$ is higher in the doped sample than in the pristine one. Above 10^4^ Hz, we can consider that the doped sample is exclusively resistive (instead of capacitive) as its ionic conductivity $\sigma^{\prime}$ remains independent of the frequency and capped at 1 mS cm^−1^ (Fig. 2e). This result is correlated by the phase angle *Φ* increasing from 10^3^ Hz and approaches 0° at 10^5^ Hz as well as the real impedance $Z^{\prime }$ values that dominates over the imaginary ones $Z^{\prime \prime }$ (Fig. S8a).

In terms of permittivity, the peak observed on the dielectric loss $\varepsilon^{\prime\prime}$ at 10^3^ Hz (Fig. 2i) suggests the relaxation of an ionic species, which can thus correspond to either the TBA^+^ or the OH^−^ confined in the ZIF-8 pores. This ion diffusion however seems to be a combination of semi-infinite (bulk) diffusion, probably stemming from a small amount of charge carriers out with of the MOF channels, possibly TBAH remained after washing the doped sample, and mainly a finite diffusion occurring under the highly confined environment of the ZIF-8 pores. This is confirmed by the phase angle *Φ* of − 70° below 10^3^ Hz (purely semi-infinite diffusion typically exhibits − 50 < *Φ* < − 10°) and the fact that imaginary impedance values $Z^{\prime \prime }$ are also 10 times higher than the real $Z^{\prime }$ values below 10^4^ Hz (Fig. S8a). Thus, we can conclude that although there is a slim possibility for some ex-pore TBAH contribution to the overall ion conductivity of the material, this contribution remains insignificant, and the sample’s conductivity is mostly directed by the in-pore confinement providing high OH^−^. This is also in line with our FTIR results revealing a negligible amount of TBAH outside of the MOF channels.

**Figure 3:**
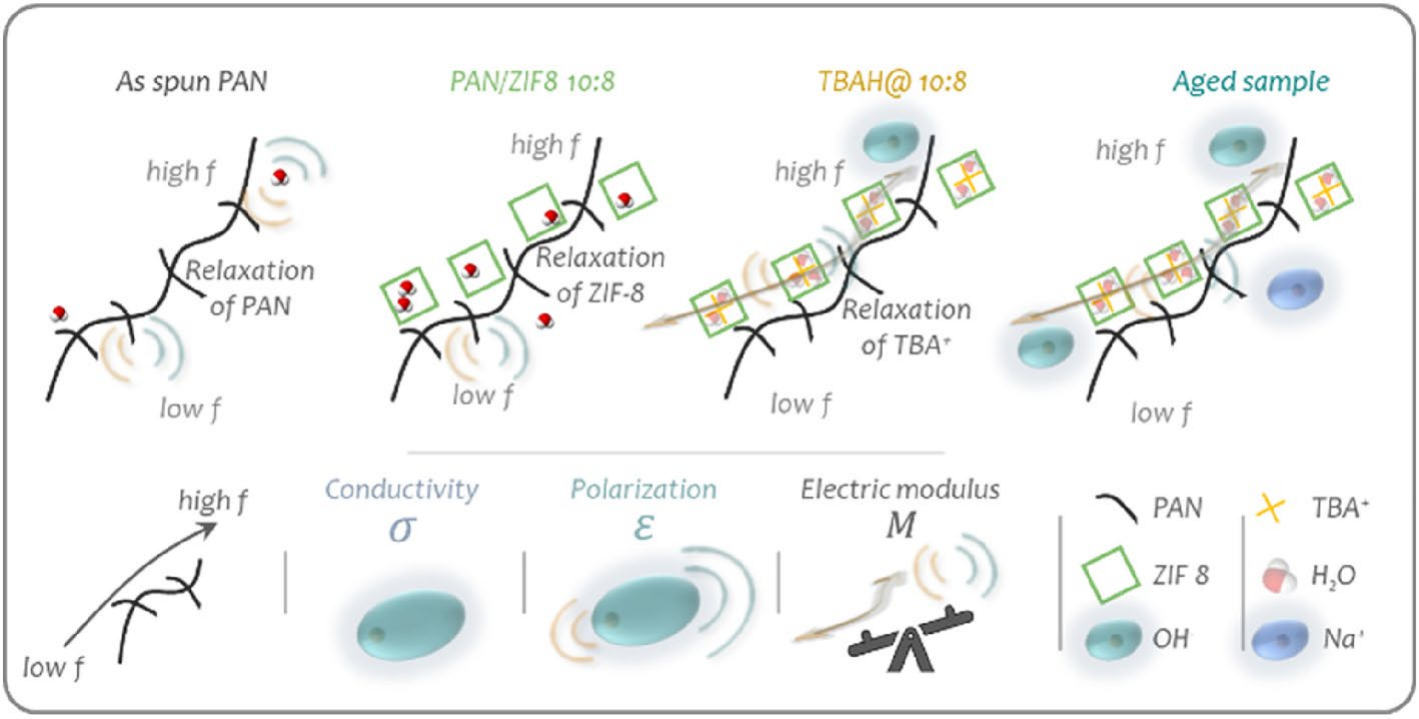
Schematic response of the fibre mats to the applied electric field during electrochemical Impedance Measurements (EIS) at low and high frequency *f* displaying polarisation and conduction effects.

According to these results, doping the ZIF-8 pores with TBAH clearly improves the MOF’s conductivity (Fig. 3). We also find that this improvement depends on the PAN:ZIF-8 ratio (Fig. S7). Interestingly, this effect follows the exact trend observed for the elastic modulus measured from nanoindentation on varying PAN:ZIF-8 proportions (Fig. S6). The ion conductivity and the elastic modulus both decrease from 10:6 to 10:8 ratio to then increase again from a 10:8 to a 10:10 ratio. We tentatively attribute this observation to the percolation of the ZIF-8 crystals inside the PAN nanofibres. This hypothesis would indicate that at lower mass ratios (10:6 and 10:8) the ZIF-8 crystals are separated by polymer chains to a greater extent. When the mass ratio increases (10:10), the ZIF-8 crystallites become in a more intimate contact with each other, forming a continuous conductive path for OH^−^ ions.

Importantly, in order for the ion-exchange membranes to be integrated in AAEMFCs, they need to display chemical robustness in alkaline conditions in addition to the mechanical robustness we already discussed. To test this, the doped fibre mats were soaked in a 2 mol dm^−3^ methanolic sodium hydroxide solution for 10 days at room temperature under inert atmosphere. We note that these ‘ageing’ conditions were chosen such as to avoid the formation of carbonate ions. The integrity of the ZIF-8 framework was maintained as shown in the XRD patterns (Fig. S9a) and the morphology of the fibre mats was not noticeably affected as seen in the scanning electron micrographs, despite the likelihood of alkaline hydrolysis of polyacrylonitrile (Fig. S9b,c). However, particles filling the inter-nanofibre voids were observed after the stability test. The diffractions peaks observed on the aged samples (Fig. S9a) suggest that, despite a thorough washing and drying with methanol, excess sodium hydroxide remained in the voids (and likely within the channels) of the PAN:ZIF-8 composite nanofibres. Taking experimental error and uncertainties into consideration, we still observe that the PAN-ZIF-8 ratio has a non-negligible influence on the ion conductivity of the aged samples and an optimum OH^−^ conductivity was found for the 10:8 ratio. We think this mechanism could be better understood by a future investigation of the distribution of ZIF-8 nanocrystals within the PAN nanofibres and the effect of percolation on the conductivity pathways of hydroxide ions.

After the above aging process, the ZIF-8 structure remains stable as seen by the unchanged Zn 2*p* peaks on the XPS spectra (Fig. S2), and overall PXRD traces (Fig. S9)^38^. It should be noted however, that our N 1*s* XPS spectrum suggests a lower amount of TBAH present in the sample, which is in line with the (002) and (001) Bragg peaks’ intensity ratio being much closer to that of the powdered ZIF-8, suggesting a lower degree of pore loading (Fig. S9b). This suggests that some TBAH would have leached out from the fibre mats on aging, as opposed to what was observed by N_2_ adsorption previously under similar ageing conditions^26^.

After stability testing, the ion conductivity of PAN:ZIF-8 10:8 further improved to 20 mS cm^−1^ at ambient-temperature (~ 22 °C). This result, however, cannot be solely attributed to an increased concentration of unbonded ions in the MOF channels as the amount of TBAH dopant was reduced. PXRD data on the aged sample indeed shows the presence of NaOH crystals in the fibre mats (Fig. S9), which would imply the presence of NaOH outwith of the MOF pores. As the NaOH conductivity in its supersaturated state approximates 100 mS cm^−1^^50^, the measured high ion conductivity therefore must have a significant contribution from NaOH. It should also be noted that in the case of the NaOH additive, neither the cation nor the anion is anchored and therefore both are expected to contribute to the total ion conductivity. The increased charge-carrier concentration is characterised by a higher dielectric constant $\varepsilon^{\prime}$, with however low values of electric modulus $M^{\prime }$ ensuring that conductivity dominates over dielectric effects. The lower relaxation time (peak on the dielectric loss $\varepsilon^{\prime\prime}$) of the aged fibre mat compared to the TBAH-doped one, could be explained by the higher charge concentration due to the high amount of ions from the NaOH additive in the aged sample (Fig. 2i). The imaginary conductivity $\sigma^{\prime \prime }$ shows for both the doped and aged membrane a similar peak indicating similar tendency for collision-related energy-loss (Fig. 2h). These two results suggest that the average inter-molecular distances are similar in the TBAH-doped ZIF-8 with a highly confined occupancy of the ionic species, and the pore and interfibrous void filling by NaOH in the aged sample. This in turn further confirms the molecular confinement in the doped sample and explain its particularly high ion conductivity of 1 mS cm^−1^ at room temperature.

The total ion conductivity of 21.6 mS cm^−1^ measured at room temperature for the aged fibre mats exceeds those previously published reporting the use of similar techniques^21^. The powdered ZIF-8 combined with NaOH, used as a reference for this work, showed an ion conductivity of 2.3·10^−6^ mS cm^−1^ in high relative humidity (90%) and at room temperature^26^. For a high degree of confidence, we derived conductivity values (Fig. 2) using two different approaches, which fully coincide delivering evidence that the calculation methods are robust.

Our approach for producing adaptable porous free-standing hydroxide-ion conducting films based on MOFs, presented herein, has proven to be very competitive for two main reasons: (i) the ion conductivity of the doped composite membranes is several orders of magnitude higher than the empty ZIF-8 powders^26^ and post stability testing we indeed measured a record-high ambient-temperature hydroxide-ion conductivity for MOF-based specimens, which is comparable with data obtained for other OH^−^ conducting MOF-based specimens at higher temperatures or in liquid electrolytes^13,19,20,51^; and (ii) the nanofibre mats exhibit high flexibility and can be easily handled, which highlights their future potential as hydroxide-ion exchange materials.

We would like to make a final remark concerning the potential of our approach presented in this work for integration in real AAEM electrolysers and fuel cell devices. Crossover of gases must be avoided to ensure safety and efficiency. We would like to point out that this is a requirement which is underexplored when it comes to MOF-based fibre mats. We recognise that this is crucial to avoid crossover and we propose that such approach for film synthesis which may be adapted for limiting its impact.

Observing the SEM micrographs of the as-spun samples (Fig. 1), it is apparent that the electrospun mats contain a high volume-fraction of void spaces between the fibres, which would likely allow for crossover. For polymer membranes, a typical strategy to lessen this effect would be to increase the film thickness. In the case of our electrospinning approach the porosity of the mats usually approximates 90% and an increase in thickness is unlikely to avoid hydrogen crossover as the inter-fibre pore size would remain of micron size. Therefore we stipulate two approaches: (i) a densification of the fibrous network by compression of the membranes to reduce the void fraction and pore size as shown in recent literature^13,52^; (ii) a filling of the void with MOF particles. As we have previously shown that the major ion-conductivity pathway is through the MOF channels we have opted for the latter approach, thus the inter-fibrous voids of the composite PAN:ZIF-8 mats were filled with ZIF-8 particles. Nanofibrous mats may be ‘coated’ with metal–organic frameworks in such a way that the nascent MOF particles preferentially form within the inter-fibrous voids. Such an approach makes use of seeding of the MOF structure, i.e. using the MOF crystallites integrated in the fibres as nucleation sites. In essence, the MOF-based nanofibre mats are immersed in the MOF synthesis mixture during the entire reaction duration. Henceforth we refer to this process as ‘coating’. We explored this approach by performing one (1C) or two (2C) subsequent coating rounds.

As expected, the coated specimens display a much denser morphology than the ‘uncoated’ fibre mats, as the ZIF-8 crystallites fill the microscopic inter-fibre voids (Fig. S10). We note that this coating also reduces their flexibility, as observed by the nanoindentation results (Fig. S6), without practically affecting the thickness of the membranes (Table S1). As mentioned above, such tightening could come to the detriment of the ion conductivity of the coated mats, for which reason we have carried out a set of ion conductivity measurements on undoped, doped, and aged fibre mats.

The void of the composite PAN:ZIF-8 10:8 films were filled by ZIF-8 crystallites, a composition which we selected arbitrarily but that is representative of all compositions reported herein. Interestingly, a denser coating improves the fibre mat’s ion conductivity in the absence of dopant by a factor of 10 after the first coating and 20 after the second one, reaching a conductivity of 5 mS cm^−1^ (Fig. S11), which in itself also is a record conductivity value for a pristine or empty MOF^14,26,53^. This can be explained by an easier hydration of the coated specimens (fewer hard-to-wet larger voids) compared to the uncoated composite ones where the ZIF-8 is embedded in the hydrophobic PAN polymer, which underlines that the primary location of ion conductivity is within the MOF channels. Thus, in the absence of dopant, the amount of ZIF-8 within the fibre mats strongly affects ion conductivity.

However, doping the coated samples (1C, 2C) has an adverse effect on the OH^−^ conductivity (Fig. S11), see Nyquist plots and impedance values (Figs. S12 and S14). This phenomenon could be attributed to the lower orientation of the denser ZIF-8 layer, whose PXRD pattern is more similar to the totally random crystallite orientation, observed for the powdered sample, than to that of the ZIF-8 containing fibre mats, and which, as seen earlier, is less conductive than the samples featuring some degree of crystallite orientation (Fig. S13). In summary, we show that the density for our PAN:ZIF-8 electrospun fibre mats may be controlled through further void filling, or coating, however in the case of the doped sample, this also lowers the ion conductivity, which we rationalise with the more random orientation of the MOF crystallites.

**Figure 4:**
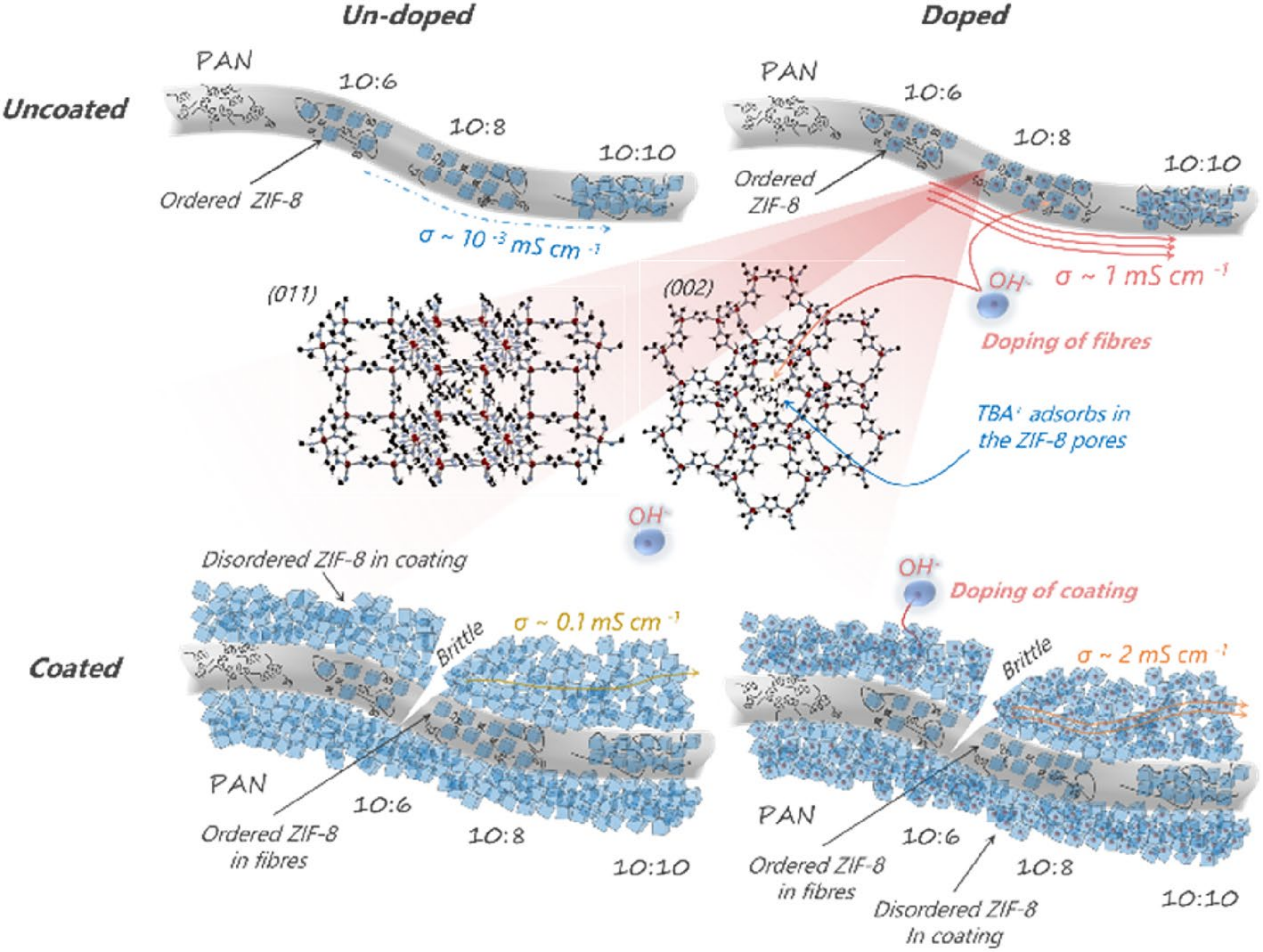
Scheme of the membranes after coating and doping showing the ordered ZIF-8 within the PAN nanofibres and the disordered ZIF-8 in the coating.

Finally, we sum up the findings of this study in Fig. 4. The electrospinning of PAN-ZIF-8 solutions aligns the ZIF-8 crystallites which improves their OH^−^ conductivity. Further doping with TBAH increases the conductivity up to 1 mS cm^−1^, while after an ageing process in alkaline solution a record conductivity of 21.6 mS cm^−1^ has been measured for ion-conducting polymer free MOF-based materials at room temperature, while maintaining flexibility (albeit this figure has a significant contribution from NaOH). The composite mats coated with ZIF-8 display random orientation of MOF crystallites, which decreases the impact of the doping on the OH^−^ conductivity, while making the fibre mat less permeable but at the same time more brittle.

## Conclusions

We report a new class of adaptable free-standing hydroxide-ion exchange fibre mats synthesised by electrospinning PAN:ZIF-8 nanofibres and doping of tetrabutylammonium hydroxide on the ZIF-8 pores, for a targeted use in alkaline anion exchange fuel cells and electrolysers. While polyacrylonitrile serves as a flexible and chemically robust substrate, the MOF creates a porous framework to host and conduct OH^−^ through its nanoporous channels. The immobilisation of tetrabutylammonium ions in the nanoporous ZIF-8 fastens the charge transfer of OH^−^ diffusion kinetics by a combination of confinement and improved hydrophilicity. Our study shows that the electrospinning partially aligns the ZIF-8 crystals allowing an ion conductivity in the fibre mats six orders of magnitude higher than that afforded by the powdered samples observed previously^26^. This shows that direct co-electrospinning of a polymer:MOF solution does not necessarily limit the accessibility of the MOF to ions as it had been suggested^16^. Furthermore, we have evaluated the stability of the so-obtained intrinsically hydroxide-ion conducting fibre mats in alkaline solution to find that no significant deterioration in structure and composition may be observed. The ion conducting performance of the membranes is even improved, which we rationalise in terms of the increased hydroxide-ion concentration. In fact, after the stability testing, we obtain a record-high room temperature OH^−^ conductivity of 21.6 mS cm^−1^ for a metal–organic framework containing mobile OH^−^ ion. These are preliminary results as the permeability of the porous self-supporting films and their resistance in strong alkaline conditions over time at various potentials should be tested in future studies, nevertheless our results are in line with a recent literature analysis highlighting the effect of processing on both ion conductivity and MOF stability^8^. Given the facile and relatively low-cost procedure, these remarkable results will help the rational design of high-ion-conductivity, low-cost, robust and flexible MOF-based fibre mats at room temperature and thus facilitate the penetration of alkaline anion exchange membranes onto the market.

## Materials and methods

### Chemicals

Zinc nitrate hexahydrate (Zn(NO_3_)_2_·6H_2_O) reagents grade 98%, methanol (MeOH) anhydrous 99,8%, *N*,*N*-dimethylacetamide (DMAc) anhydrous 99,8%, polyacrylonitrile (PAN) (*M*_w_ ~ 150 000 g mol^−1^), tetrabutylammonium hydroxide hydrate (NBu_4_OH·30 H_2_O, TBAH) purity > 99,0% and 2-methylimidazole (Hmim) 99% were purchased from Merck/Sigma Aldrich and used without further modification.

### Preparation

*ZIF-8 synthesis*. ZIF-8 was synthesised by mixing a methanolic solution of Zn(NO_3_)_2_·6H_2_O (10 mmol, 2.976 g dissolved in 100 cm^3^ of methanol) and 2-methylimidazole (Hmim) (40 mmol, 3.284 g dissolved in 100 cm^3^ of methanol), according to a method outlined previously^26^ (Table 1). The mixture was left at room temperature for 24 h without stirring and a white precipitate was collected. The precipitate was filtrated and washed with methanol then dried.

**Table 1:** Experimental parameters preparation of ZIF-8.

Compounds	Zn(NO_3_)_2_·6H_2_O	2-methylimidazole (Hmim)
Molar mass (g mol^−1^)	297,482	8210
Mass (g)	2976	3284
Number of moles (mmol)	10	40
Molar equivalent n (–)	1	4

*Electrospinning.* Various solutions of a PAN:ZIF-8 mixture (see mass ratios in Table 2) were prepared in *N*,*N*-dimethylacetamide. ZIF-8 was ground using a mortar and pestle and added to *N*,*N*-dimethylacetamide with vigorous stirring before being added to the PAN precursor solution. The mixtures were stirred at 50 °C overnight to obtain translucent solutions. The mixtures were then electrospun in a chamber (Nanobox, Plaslab) where the temperature was maintained at 22 ± 0.7 °C and the relative humidity at 25% ± 3%. The mixtures were electrospun at a rate of 1 cm^3^ per hour using an 18 Gauge needle positively charged at 25 kV and placed at 25 cm from a flat grounded aluminium plate serving as a collector for the nanofibres.

**Table 2:** Experimental parameters for the preparation of electrospun fibre mats.

Samples	Polyacrylonitrile (PAN)	ZIF-8	DMAc
PAN ZIF-8 10: 6	1 g	0.6 g	10 g
PAN ZIF-8 10: 8	1 g	0.8 g	10 g
PAN ZIF-8 10: 10	1 g	1 g	10 g

*Coating.* Coating was carried out in a bid to develop a method to hinder crossover and to probe the effect of ZIF-8 particulate density on the ion conductivity. Using the same proportions as for the ZIF-8 synthesis, Zn(NO_3_)_2_·6H_2_O (5 mmol, 1.488 g dissolved in 50 cm^3^ of methanol) and 2-methylimidazole (Hmim) (20 mmol, 1.642 g dissolved in 50 cm^3^ of methanol) were vigorous stirred for 10 min. The PAN:ZIF-8 mats were then soaked in the solution at 60 °C for at least 12 h. They were then cooled down to room temperature and washed three times with MeOH before drying at room temperature. A second and third coating (samples 2C, 3C) could be achieved by repeating the same procedure. However, it was found that three coatings induced a significant loss of flexibility, which made them unsuitable for further experiments.

*TBAH doping.* The electrospun fibre mats were soaked at 60 °C for 24 h in a solution of TBAH·30H_2_O dissolved in methanol. The doped mats were then thoroughly washed with methanol and kept under nitrogen atmosphere to avoid carbonation of the hydroxide ions (Table 3).

**Table 3:** Experimental parameters for the doping of the fibre mats.

Compounds	PAN:ZIF-8	TBAH·30 H_2_O	MeOH
Molar mass (g mol^−1^)	227.58	799.93	
Mass (g)	30.8	627.73	
Number of moles (mol)	0.135	0.785	
Molar equivalent n (–)	1	5.8	
Volume (mL)			32

*Stability testing.* The prepared nanofibre mats were first vacuum dried and stored under inert condition. They were then soaked for 10 days at room temperature in a 2 mol dm^−3^ methanolic sodium hydroxide solution prepared using anhydrous methanol (MeOH) and degassed NaOH. After soaking, they were washed three times with anhydrous MeOH then dried under vacuum at room temperature in a desiccator.

### Materials characterisation

*Powder X-ray diffraction (PXRD)* patterns were collected on a Panalytical X’Pert Pro in reflection mode using a Cu *K*_*α*_ anode (*λ* = 1.54178 Å), divergence slit, Ni-filter and a range of 5–120° 2*Θ*.

*Fourier-transformed infrared (FTIR) spectroscopy* was carried out using an attenuated total reflectance (ATR) setup on a Bruker Tensor 27 spectrometer. A resolution of 4 cm^−1^ was used with a range of 400–4000 cm^−1^, with 32 scans. The OPUS software was used to for spectrum acquisition. The TBAH and PAN:ZIF-8 10:8 spectra were respectively normalised on the 2875 cm^−1^ and 2242 cm^−1^ peak of the TBAH@PAN:ZIF-8 10:8 spectra.

*Scanning electron micrographs (SEM)* were acquired on a FEI Phenom instrument using an acceleration voltage of 5 kV and a working distance of 12 mm. The samples were sputtered with Au using pulsed laser deposition at a current of 15 mA for 30 s to improve the materials conductivity for imaging.

*Nanoindentation* experiments were performed using an ultra-micro-indentation system UMIS-2000 CSIRO equipped with a spherical ruby indenter (diameter 1 mm) placed perpendicular to the samples. The maximum load applied to the samples was set to 5 mN. To ensure a representative distribution of values, 10 indents were performed on each sample separated by a minimum distance of 0.1 mm. The temperature and humidity were not actively controlled and ranged respectively between 20 and 25 °C and 40–50% during the experiments.

#### Calculation of the elastic modulus from nanoindentation

1 $$\begin{array}{*{20}c} {E^{\prime} = \sqrt {\frac{{F^{2} \cdot D^{ - 1} }}{L \cdot A}} \left[ {{\text{N}}\,{\text{m}}^{ - 2} } \right]} \\ \end{array}$$

where $F$, $D, L$ and $A$ represent the force applied [N], the displacement [m], the length of the thickness [µm] and nanomat area [cm^2^], respectively.

*X-ray photoelectron spectra (XPS)* were acquired under ultrahigh vacuum (< 5 × 10^−7^ Pa) on a Thermo Fisher Nexsa XPS System with a monochromated Al *K*_α_ X-ray source (photon energy = 1486.7 eV). A pass energy of 50 eV was used with a flood gun as an electron source for charge neutralisation. We would like to note that on account of the highly complex nature of the C 1*s* spectra, several mathematically possible spectral fits also were physically meaningful. For this reason, we deemed that quantitative evaluation of the data, including calibration, would be too speculative, and we are restricting ourselves to a qualitative analysis. It should be also noted that XPS spectra were acquired after the rest of the analysis and the samples were kept in air, for this reason, it is likely that particularly the more hydrophilic samples both adsorbed moisture and the aged (NaOH-treated) samples reacted with atmospheric CO_2_ forming carbonate ions.

#### EIS measurements and ion conductivity evaluations

The self-supporting porous films (0.5 cm radius) were compressed in a perfluoroalkoxy alkane (PFA) *Swagelok ½”* union assembly using two low-corrosion stainless steel (*Hastelloy)* current collectors (½” diameter) to form a two-electrode probe and measure the through-plane conductivity. Electrochemical Impedance spectroscopy (EIS) measurements were performed on a Gamry potentiostat operated at an amplitude of 10 mV in the frequency range from 1 MHz to 100 mHz under open circuit potential conditions. All measurements were carried out inside a glove bag under ambient temperature (22 ± 3 °C) and relative humidity (*ca.* 95%) with overnight nitrogen purge before and during measurements to maintain a stable nitrogen environment and ultra-low CO_2_ levels. Open circuit potential was monitored for 200 s prior to each measurement to ensure stable connectivity between the specimens and current collectors.

To improve the reliability of our results, the electrical/ionic conductivities of the mats were calculated using two independent methods. We first used the charge transfer resistance to provide a conductivity value independent of the frequency (Eq. 2). This charge transfer resistance, so-called bulk resistance (*R*_*b*_), of each fibre mat was estimated from the measured Bode and Nyquist impedance plots using respectively the *θ*_*max*_ (Eq. 3) and the *Nyquist Semicircle* method (Fig. S7)^54^. Both methods and their related errors estimated on the log-derivative method are presented in Eq. (3).

The measured OH^−^ conductivity (*σ*) in the unit of mS cm^−1^ was calculated using the charge transfer resistance as the following:

2 $$\begin{array}{*{20}c} {\sigma = \frac{t}{{R_{b} \cdot A}}} \\ \end{array}$$

where $t$ represents the membrane thickness (µm), ${R}_{b}$ the charge transfer resistance $(\Omega$) and A the specimen area (cm^2^).

The charge transfer resistance ${R}_{b}$ value was estimated using the two following methods:

- $\theta_{max}$ *method* uses the impedance observed at highest phase angle in the Bode plot, which is noted $\theta_{max}$.
  3 $$\begin{array}{*{20}c} {R_{b} \left( {\theta = \theta_{max} } \right) = cos\left( {\theta_{max} } \right)*\frac{\pi }{180\left(^\circ \right)}*\left| Z \right|_{{\theta_{max} }} } \\ \end{array}$$

where $\theta_{max}$ is in degrees and $\left| Z \right|$ is the impedance modulus in Ohms.

- *Nyquist Semicircle method* extrapolates the charge-transfer semicircle observed in the Nyquist plot to obtain the intersection with the real axis of impedance observed at low frequency). If the Nyquist plot does not show a clear partial semi-circular shape, the linear region was extrapolated and the charge transfer resistance was read at the intersection with the *x*-axis (see Fig. S3).

The error of the thickness measurement, diameter, electrode area and ${R}_{b}$ values was estimated using the log-derivative method using the following equation:

4 $$\begin{array}{*{20}c} {\frac{\Delta \sigma }{\sigma } = \frac{\Delta t}{t} + \frac{\Delta A}{A} + \frac{{\Delta R_{b} }}{{R_{b} }}\; \left[ \% \right]} \\ \end{array}$$

$\frac{\Delta t}{t}$ can be determined by estimating the error of the thickness measurement (precision of the caliper), usually around 5–20%. $\frac{\Delta A}{A}$ can be determined by estimating the error of diameter *D* (precision of the calliper), usually around 5%; If the electrode is a rectangle, then $\frac{\Delta A}{A} = \frac{\Delta side 1}{{side 1}} + \frac{\Delta side 2}{{side 2}};$ $\frac{{\Delta R_{b} }}{{R_{b} }}$ can be determined by taking the difference between the *θ*_*max*_ and the *Nyquist Semicircle* method, usually between 3 and 25%.

Complex permittivity and electric conductivity were calculated according to:

5 $$\begin{array}{*{20}c} {\varepsilon \left( \omega \right) = \varepsilon^{\prime}\left( \omega \right) + i\varepsilon^{\prime\prime}\left( \omega \right)\quad \left[ {{\text{F}}\,{\text{m}}^{ - 1} } \right]} \\ \end{array}$$

6 $$\begin{array}{*{20}c} {\sigma \left( \omega \right) = \sigma^{\prime}\left( \omega \right) + i\sigma^{\prime\prime}\left( \omega \right)\quad \left[ {{\text{S}}\,{\text{m}}^{ - 1} } \right]} \\ \end{array}$$

where $\omega = 2\pi f$ is the angular frequency and $i$ the imaginary unit, defined as ${i}^{2}=-1$.

Here, *σ* represents the mobility of the charges, ε their polarisability and M the balanced contribution of the charge transfer and the polarisability of the material in respond to the applied field^55^. The real term of conductivity, $\sigma^{\prime}$, relates the current density to the applied electric field $\overrightarrow{E}$, while the imaginary term of conductivity, $\sigma^{\prime\prime}$, represents the energy loss from inter-molecular collisions. Similarly, the dielectric constant $\varepsilon^{\prime}$ represents the ability of the material to screen the electric field by polarisation of bound charges in dipoles, and the imaginary part called loss factor $\varepsilon^{\prime\prime}$ represents the energy loss from inelastic scattering as in Eq. (8) below.

Using the impedance data obtained from the EIS measurements, the electrical permittivity $\varepsilon \left(\omega \right)$ and conductivity $\sigma \left(\omega \right)$ can be formalised as follows:

7 $$\begin{array}{*{20}c} {\varepsilon \left( \omega \right) = \frac{t}{{A\omega \varepsilon_{0} }} \cdot \frac{{Z^{\prime}\left( \omega \right)}}{{\left| Z \right|^{2} }} + i\frac{t}{{A\omega \varepsilon_{0} }} \cdot \frac{{Z^{\prime\prime}\left( \omega \right)}}{{\left| Z \right|^{2} }}\quad \left[ {{\text{F}}\,{\text{m}}^{ - 1} } \right]} \\ \end{array}$$

8 $$\begin{array}{*{20}c} {\sigma \left( \omega \right) = \omega \varepsilon_{0} \varepsilon^{\prime\prime}\left( \omega \right) + i\omega \varepsilon_{0} \varepsilon^{\prime}\left( \omega \right)\quad \left[ {{\text{S}}\,{\text{m}}^{ - 1} } \right]} \\ \end{array}$$

9 $$\begin{array}{*{20}c} {\sigma \left( \omega \right) = \frac{t}{A} \cdot \frac{{Z^{\prime}\left( \omega \right)}}{{\left| Z \right|^{2} }} + i\frac{t}{A} \cdot \frac{{Z^{\prime\prime}\left( \omega \right)}}{{\left| Z \right|^{2} }}\quad \left[ {{\text{S}}\,{\text{m}}^{ - 1} } \right]} \\ \end{array}$$

where $\varepsilon_{0} = 8.85419\, \times \,10^{ - 12} { }\left[ {{\text{F}}\,{\text{m}}^{ - 1} } \right]$ is the vacuum permittivity (contributed capacitance to the vacuum), $t \left[ {{\text{cm}}} \right]$ and $A \left[ {{\text{cm}}^{2} } \right]$, the thickness and section of the sample.

Complex moduli and electric conductivity were calculated as follows:

10 $$\begin{array}{*{20}c} {M\left( \omega \right) = M^{\prime}\left( \omega \right) + iM^{\prime\prime}\left( \omega \right)\quad \left[ {{\text{m}}\,{\text{F}}^{ - 1} } \right]} \\ \end{array}$$

11 $$\begin{array}{*{20}c} {M\left( \omega \right) = \frac{{\varepsilon_{0} \omega A}}{t} \cdot Z^{\prime\prime}\left( \omega \right) + i \frac{{\varepsilon_{0} \omega A}}{t} \cdot Z^{\prime}\left( \omega \right)\quad \left[ {{\text{m}}\,{\text{F}}^{ - 1} } \right]} \\ \end{array}$$

### Supplementary Information

Supplementary Information.

## Data Availability

All data generated or analysed during this study are included in this published article [and its supplementary information files].
