# Supplementary material for: Development of a new class of stable and adaptable free-standing fibre mats with high room-temperature hydroxide-ion conductivity
Source: Sci Rep. 2024 Jun 24;14:14529. doi: 10.1038/s41598-024-64646-9 (PMC11196687; doi:10.1038/s41598-024-64646-9)
Supplement: Supplementary file 1 — Supplementary Information. [file 41598_2024_64646_MOESM1_ESM.docx]

SI -

Development of a new class of stable and adaptable free-standing fibre mats with high room-temperature hydroxide-ion conductivity

Servann Hérou^1^, Pauline Kasongo-Ntumba^2^, Arun Prakash Periasamy^2,3^, James King^2^, Molly McVea^b^, Szymon Doszczeczko^2^, Andy Bushby^2^, Ana Belen Jorge Sobrido^2^, Maria-Magdalena Titirici^1^, Petra Ágota Szilágyi^4*^

^1^ Department of Chemical Engineering, Imperial College Road, Kensington, London SW7 2AZ, United Kingdom

^2^ Queen Mary University of London, School of Engineering and Materials Science and Materials Research Institute, Mile End Road, E1 4NS London, UK.

^3^ Department of Chemistry, SRM Institute of Science and Technology, Kattankulathur, Tamil Nadu, 603 203, India

^4^ Centre for Materials Science and Nanotechnology (SMN), Department of Chemistry, University of Oslo, P.O. Box 1033, Blindern, N-0315 Oslo, Norway.

* Corresponding author, P.A.Szilagyi@kjemi.uio.no

***Materials characterisation***


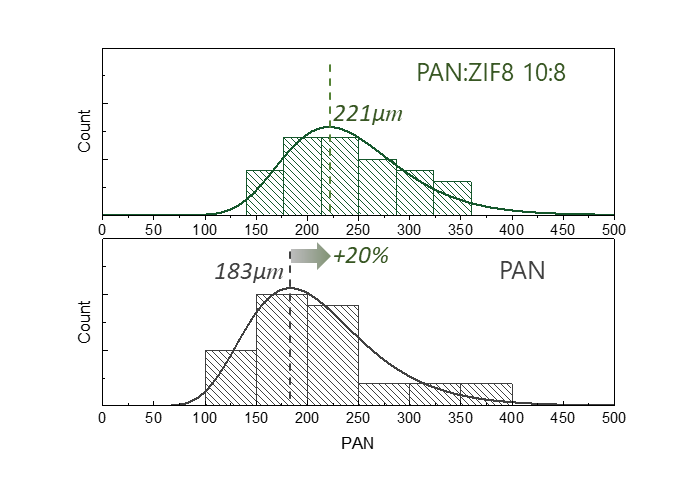


Figure S1: Diameter distribution of the nanofibres in the electrospun PAN sample and PAN:ZIF8 10:8 composite. Each diameter distribution was fitted with a lognormal curve as reported in the literature.^1^

Figure S2: X-Ray Photoelectron Spectroscopy (XPS) results showing the C 1s, N 1s, O 1s and Zn 2p, Na 1s regions and survey spectra of the different samples presented; samples are: top left PAN:ZIF-8 10:8; middle left: PAN:ZIF-8 1C; bottom left PAN; top right PAN:ZIF-8 10:8 TBAH; middle right PAN:ZIF-8 10:8 1C TBAH; and bottom right PAN:ZIF-8 10:8 TBAH NaOH.


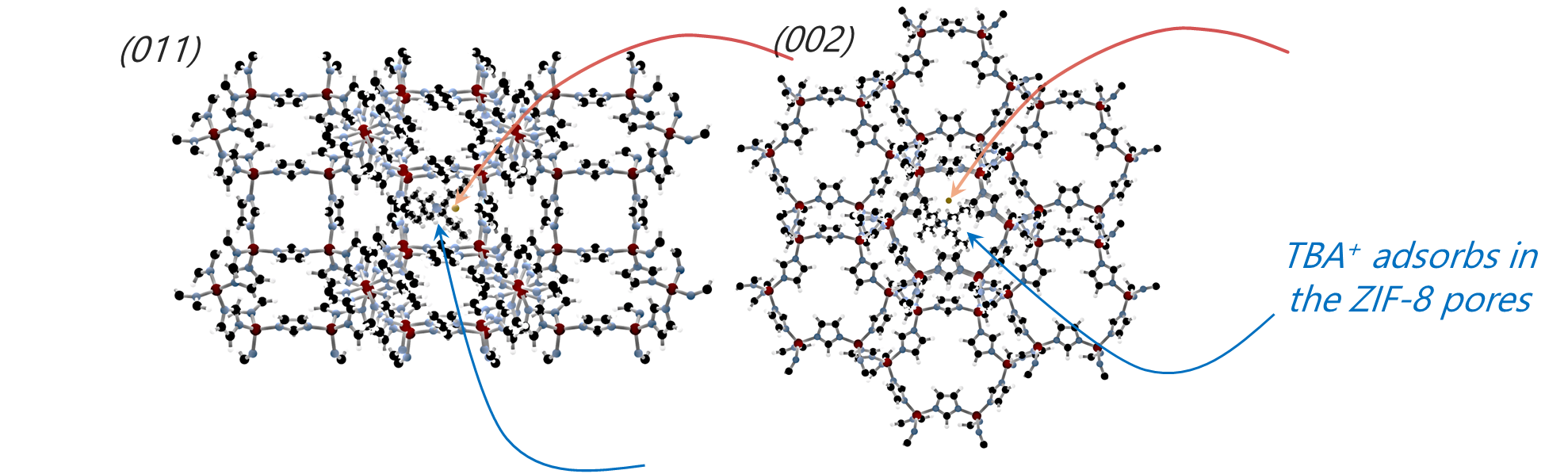


Figure S3: Representation of the (011) and the (002) planes of the ZIF-8 with the inserted TBA^+^ ions and the mobile OH^-^ in the cages of the ZIF-8


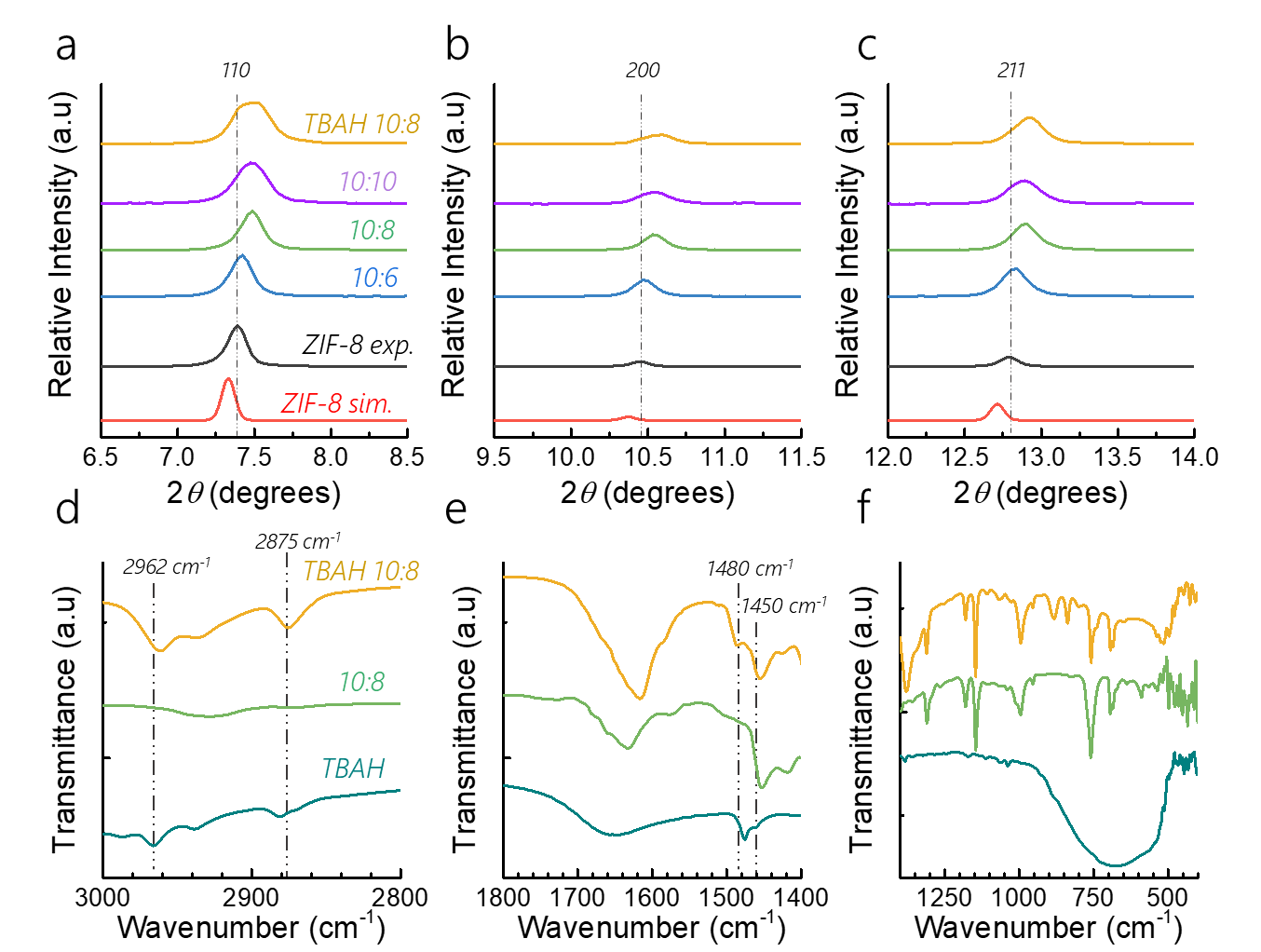


Figure S4: (a),(b) and (c) Detailed XRD patterns of simulated ZIF-8, synthesised ZIF-8, ZIF-8:PAN with ratios of 10:6, 10:8 and 10:10 and TBAH@ZIF-8:PAN 10:8. The crystallographic peaks were labelled as in the literature.^2^ (d-f) Detailed FTIR spectra of the TBAH salt, ZIF-8:PAN 10:8 and the doped analogue TBAH@ZIF-8:PAN 10:8. Transmittance was normalised.


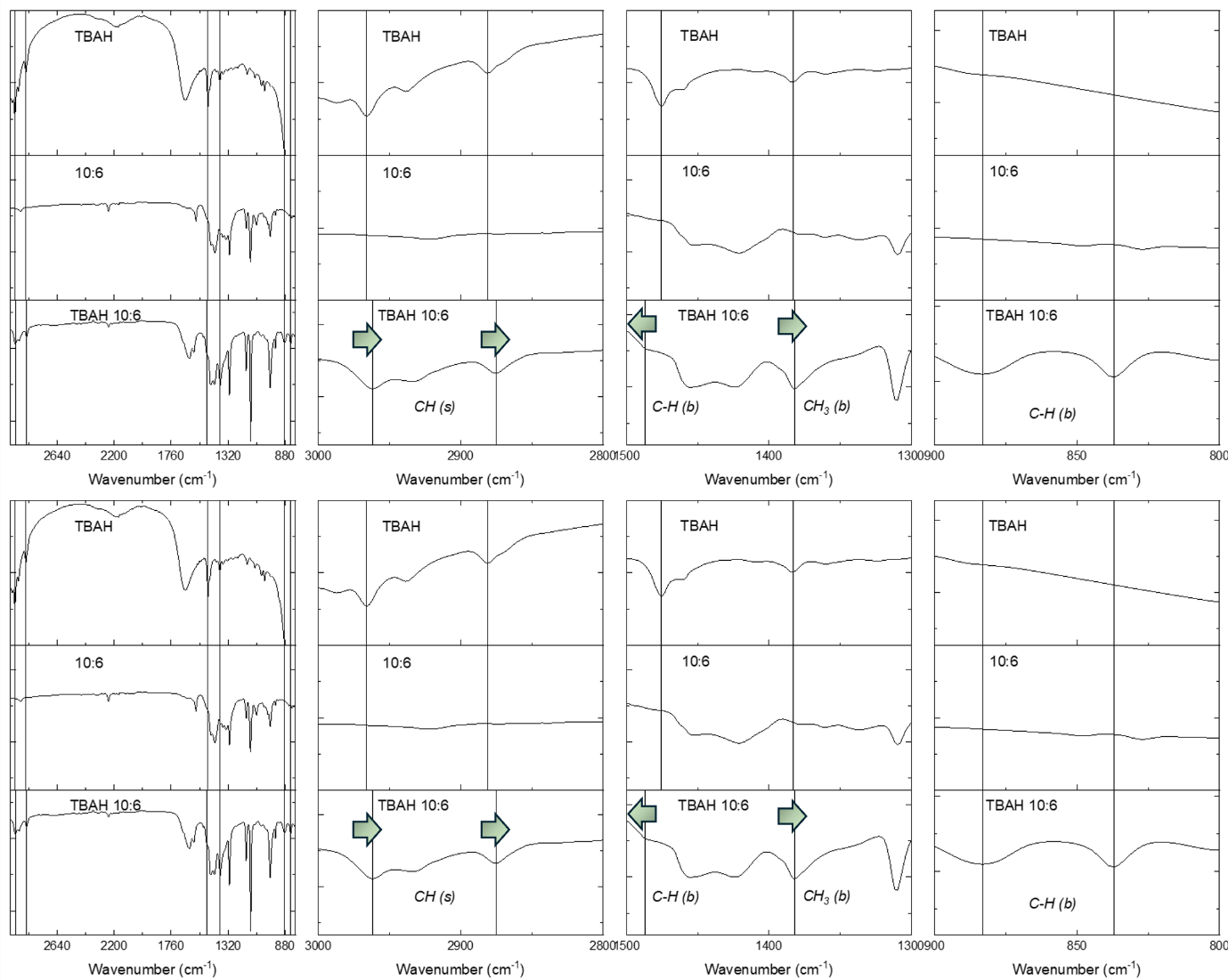


Figure S5: FTIR spectra of TBAH@ZIF-8:PAN 10:6 (top row) and 10:10 (bottom row) showing identical trends as 10:8 on compositing


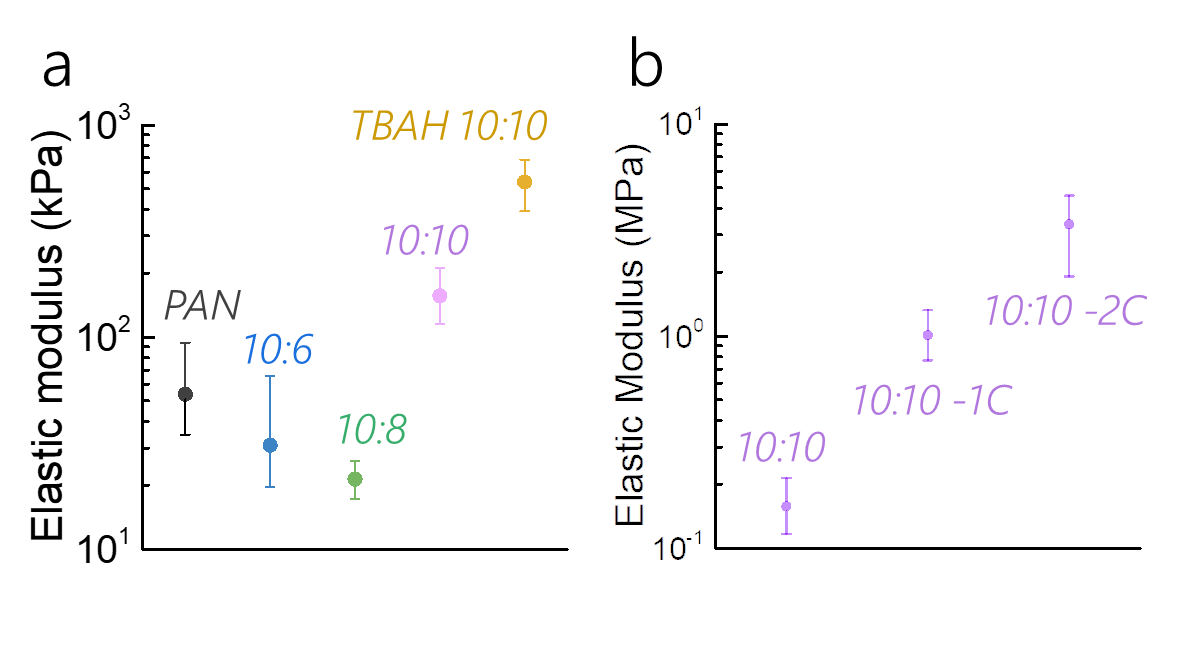


Figure S6: Nanoindentation results as (a) function of the PAN:ZIF-8 ratio and after TBAH doping and (b) function of the number of ZIF-8 coatings for 10:10.

***Electrochemical Impedance spectroscopy (EIS):***


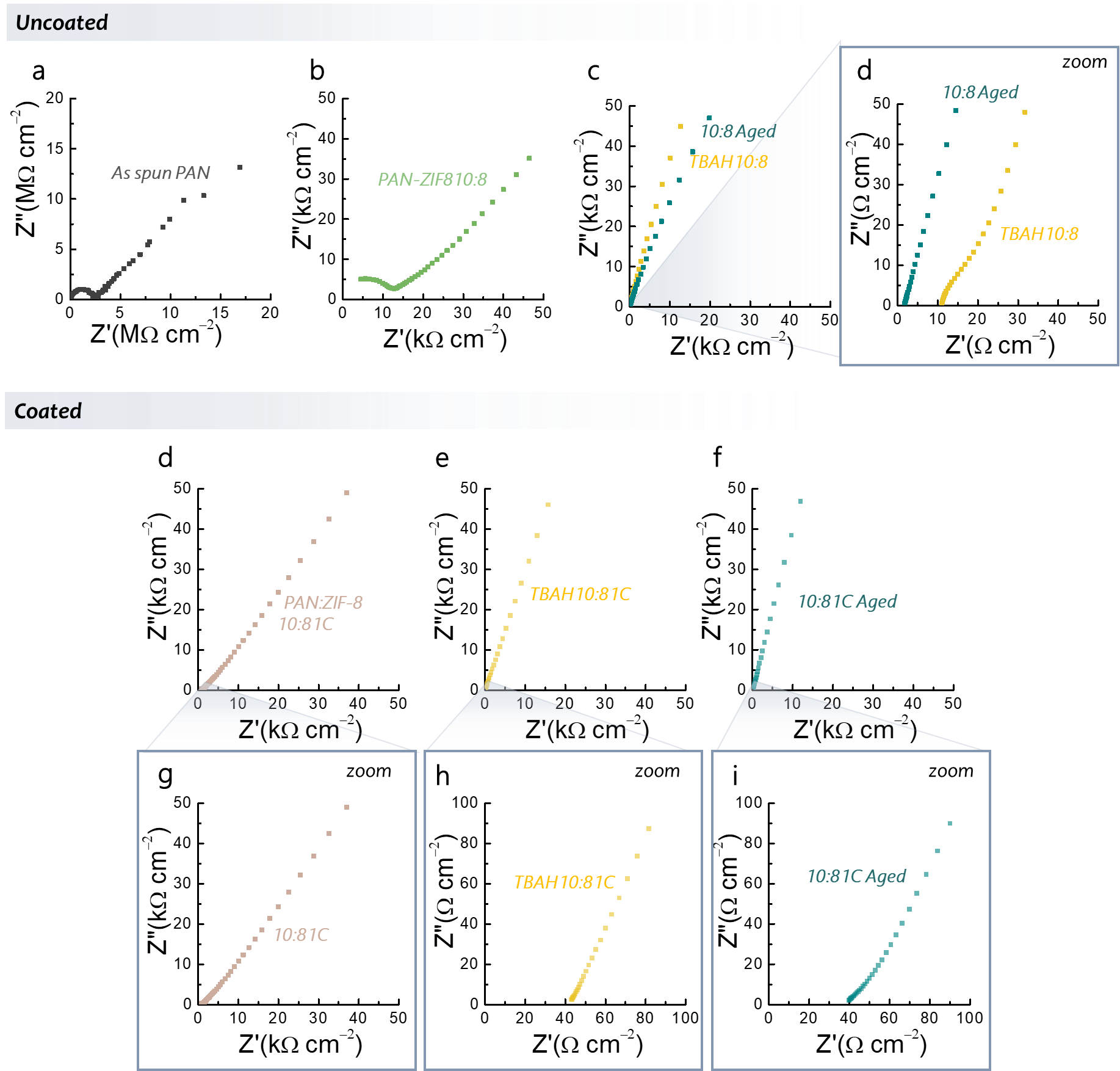


Figure S7: The ion conductivities in terms of OH^-^ ions conduction to be determined from charge-transfer resistance values derived from the linear regression of the Nyquist plots of the (a-c) uncoated samples with (c) detailed scale; (d-f) coated samples with (f) detailed scale; valuable insights could be derived from the combination of resistive and capacitive processes occurred in the respective fibre mats..


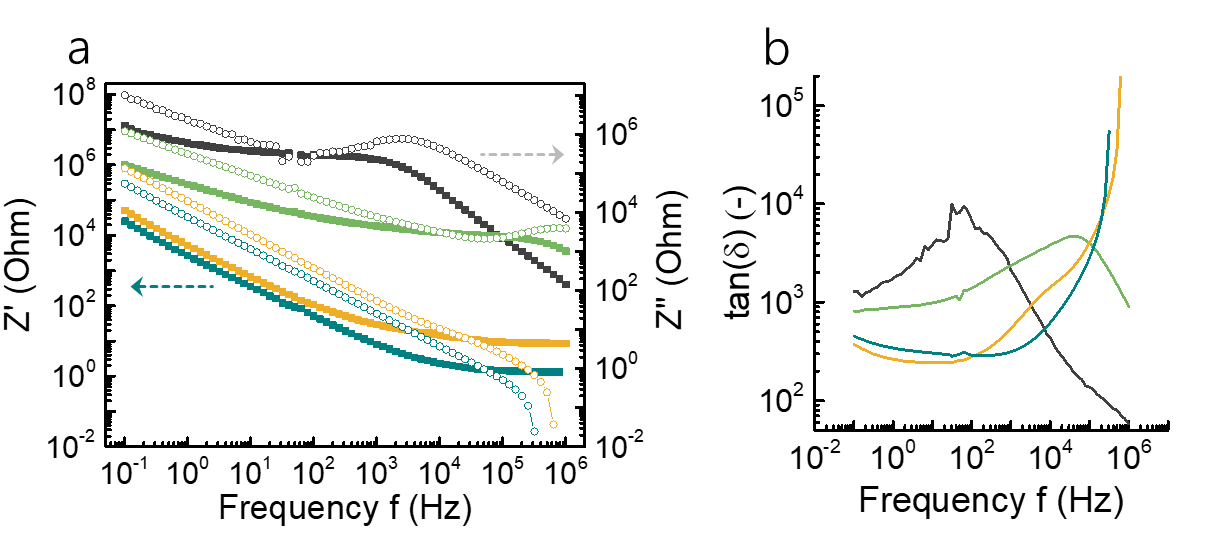


Figure S8: The upward and down-shits of the M” peaks observed with the uncoated and coated samples as a function of real parts of the impedance with changes in the frequency f (Hz) used (a); (b) peak shifts indicative of the dielectric loss, 𝐭𝐚𝐧(𝜹) observed as a function of the frequency used for the uncoated samples only.


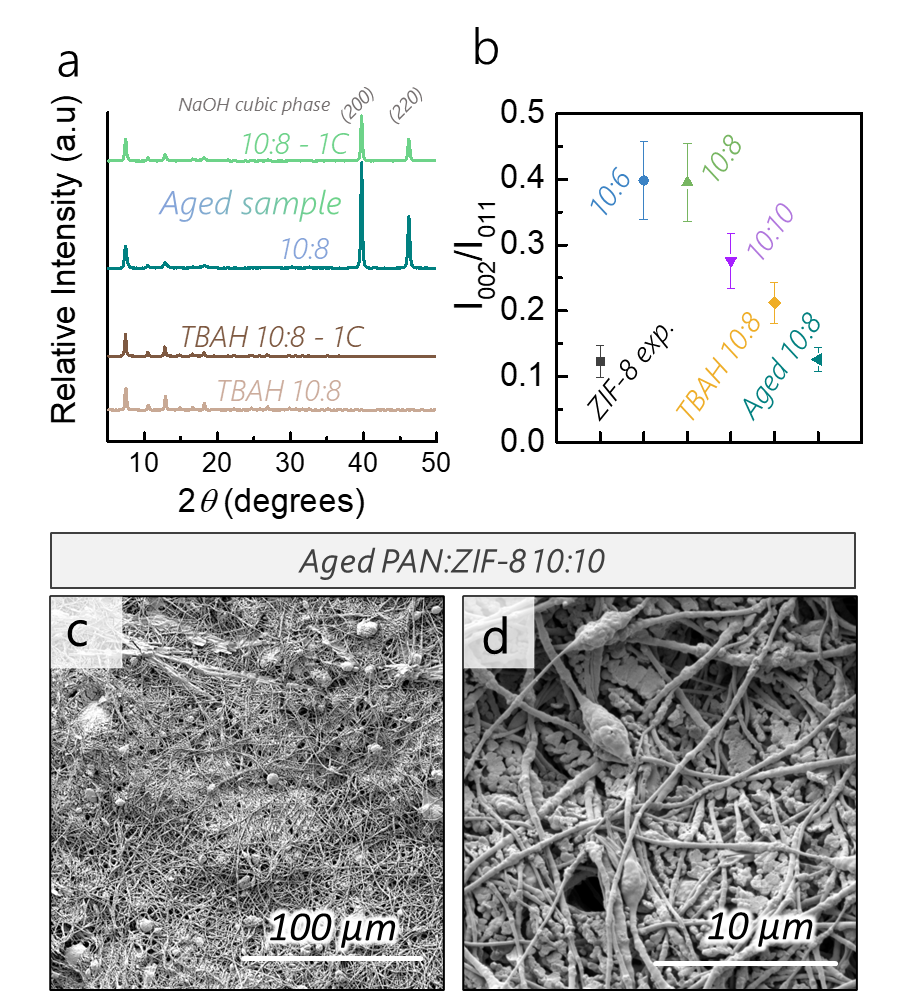


Figure S9: (a) XRD patterns of the uncoated and coated (1C) PAN:ZIF-8 10:8 after doping with TBAH and after stability testing by ageing; (b) Intensity ratios of the (002) over the (001) peak derived from the XRD patterns on Figure 1d and Figure S8a; (c,d) Scanning electron micrographs of the ZIF-8:PAN 10:10 after ageing.

**
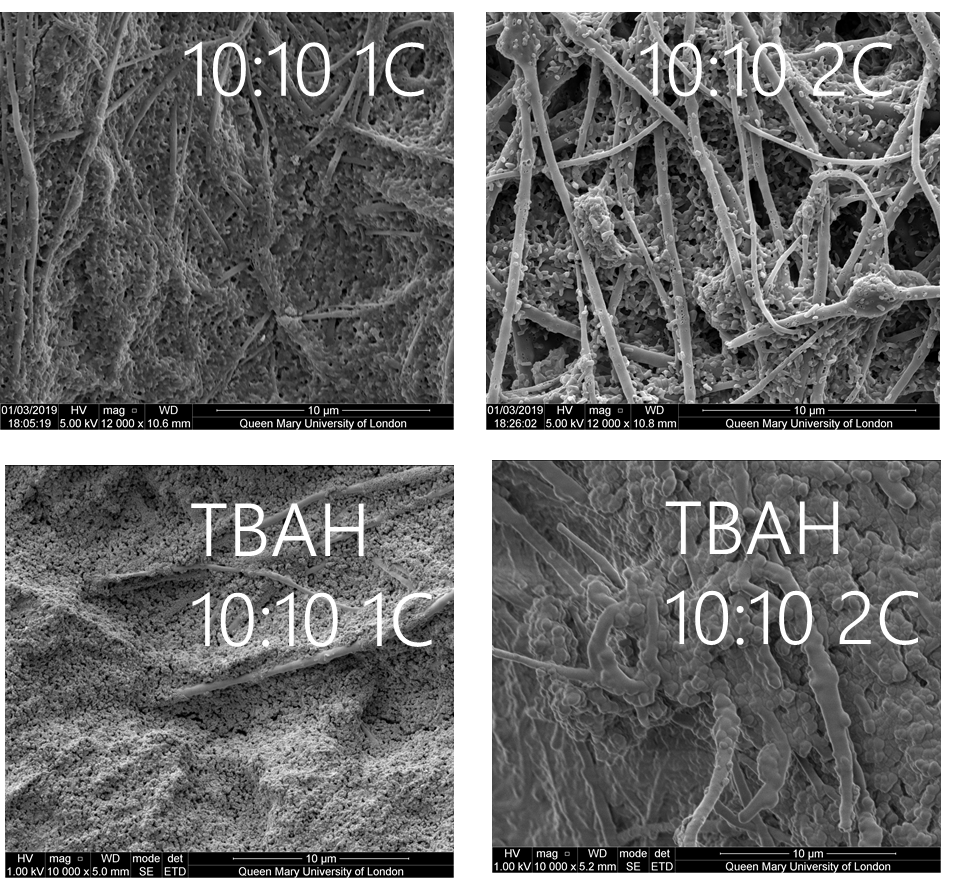
**

Figure S10: Scanning Electron Micrographs of the coated samples. ZIF-8:PAN 10:10-1C and ZIF-8:PAN 10:10-2C and their doped analogues TBAH@ZIF-8:PAN 10:10-1C and TBAH@ZIF-8:PAN 10:10-2.

Figure S11: Ion conductivities obtained via potentiostatic Electrochemical Impedance Spectroscopy (EIS) showing (a) The increased ion conductivity after the various treatments; (b) the influence of coatings on the conductivity of the undoped PAN:ZIF-8 10:8 composite membranes; (c) the influence of coatings on the TBAH doped PAN:ZIF-8 10:8 composite membranes; (d) the influence of coatings on the conductivity of the aged doped PAN:ZIF-8 10:8 composite membranes


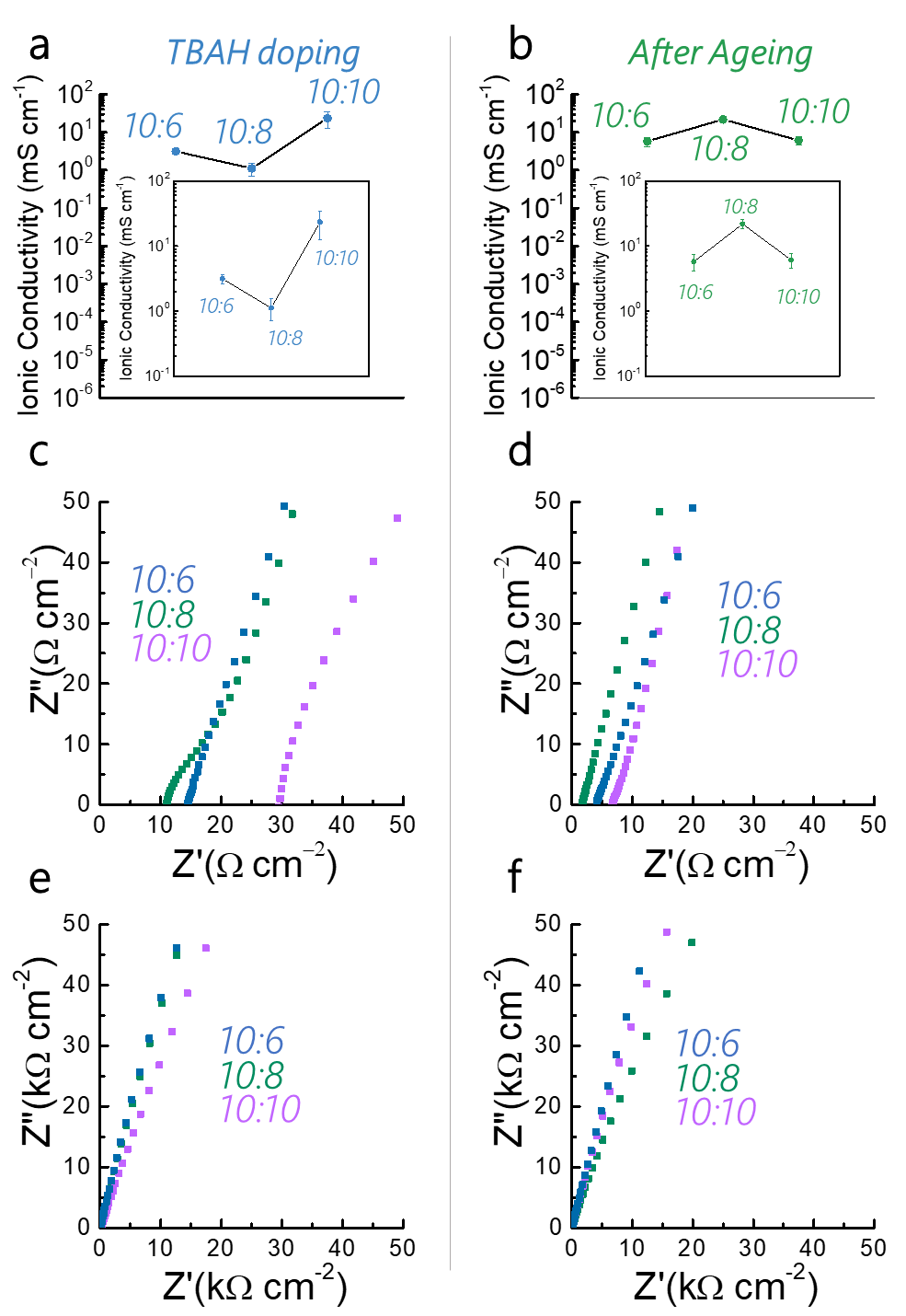


Figure S12: (a,b) Ion conductivities obtained via potentiostatic Electrochemical Impedance Spectroscopy (EIS) showing the influence of the ZIF-8:PAN ratio in the (a) TBAH doped nanofibres and (b) Aged sample; Nyquist plots showing the high frequency (c, d) and low frequency (e,f) regions for the TBAH doped (left) and aged sample membranes.


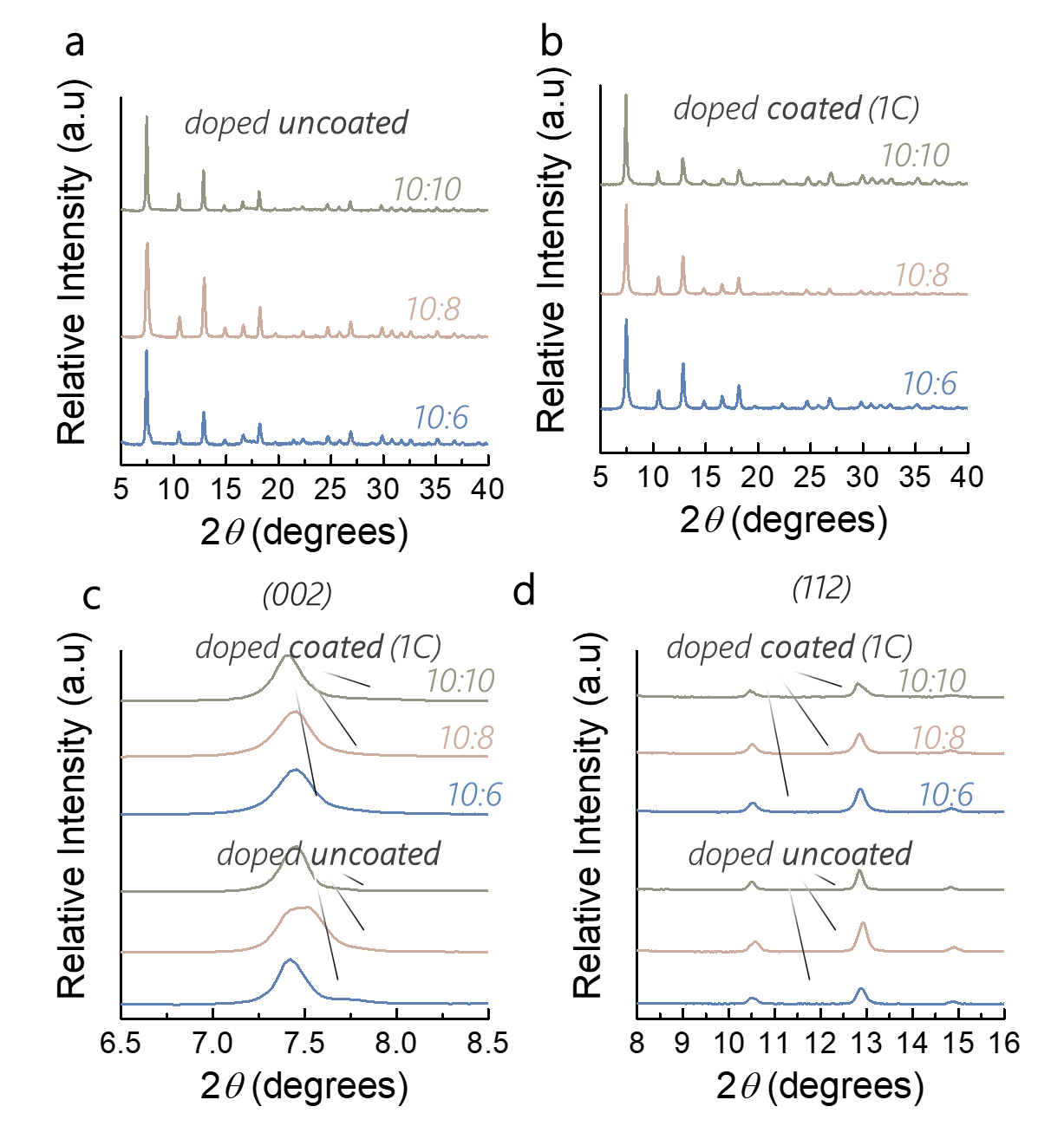


Figure S13: (a),(b) and (c) Detailed XRD patterns of ZIF-8:PAN with ratios of 10:6, 10:8 and 10:10 and their doped analogues TBAH@ZIF-8:PAN 10:6, 10:8 and 10:10. All patterns were normalised on (110) peak. The diffraction peaks were labelled as in the literature.^2^


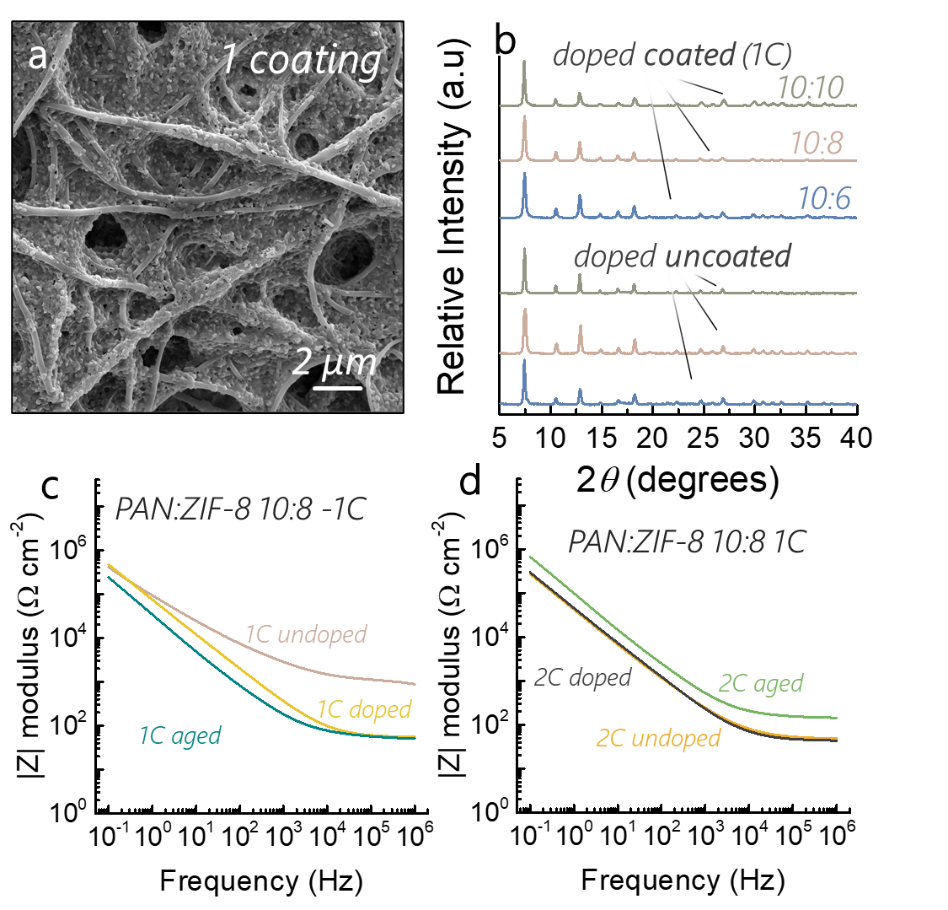


Figure S14: (a) Scanning Electron Microscope of the PAN:ZIF-8 10:8 1C; (b) X-Ray Diffractograms of various ratios of PAN:ZIF-8 in the spinning solution after 1 coating of ZIF-8 showing the similar phases of the ZIF-8 onto the fibres; Z modulus for the undoped, doped and aged PAN:ZIF-8 10:8 fibres after (c) 1 Coating -1C- and (d) 2 coatings -2C-.

**References**

(1) Hérou, S.; Crespo, M.; Schlee, P.; Luo, H.; Cristian, L.; Ro, C.; Titirici, M. The Impact of Having an Oxygen-Rich Microporous Surface in Carbon Electrodes for High-Power Aqueous Supercapacitors. *J. Energy Chem.* **2021**, *53*, 36–48. https://doi.org/10.1016/j.jechem.2020.04.068.

(2) Su, J.; Wu, W.; Li, Z.; Li, W. Self-Crystallization of Uniformly Oriented Zeolitic Imidazolate Framework Films at Air–Water Interfaces. *Dalt. Trans.* **2019**, *48*, 11196–11199. https://doi.org/10.1039/c9dt02359c.
